# Maternal Endocrine and Vascular Perturbations Reprogram the Transcriptomic and Secretory Profile of Perinatal Stem Cells: A Comparative Study of Umbilical Cord-Derived Stem Cell UC-MSCs and Human Milk-Derived Stem Cell MM-MSCs

**DOI:** 10.3390/ijms27188269

**Published:** 2026-09-17

**Authors:** Dominika Przywara, Wiktor Babiuch, Pola Kosteczko, Alicja Petniak, Adrianna Kondracka, Arkadiusz Krzyżanowski, Monika Czuba, Rafał Szymanowski, Janusz Kocki, Paulina Gil-Kulik

**Affiliations:** 1 Department of Clinical Genetics, Medical University of Lublin, 11 Radziwillowska Str., 20-080 Lublin, Poland; dprzywara17@gmail.com (D.P.); alicja.petniak@umlub.pl (A.P.); monikaczuba@umlub.pl (M.C.); rafal.szymanowski@umlub.pl (R.S.); janusz.kocki@umlub.pl (J.K.); 2 Doctoral School, Medical University of Lublin, 20-093 Lublin, Poland; 3 Student Scientific Society of Clinical Genetics, Medical University of Lublin, 20-080 Lublin, Poland; babiuchwik@gmail.com (W.B.); pola.kosteczko@wp.pl (P.K.); 4 Department of Obstetrics and Pathology of Pregnancy, Medical University of Lublin, 11 Staszica Str., 20-081 Lublin, Poland; adrianna.kondracka@umlub.pl (A.K.); arkadiusz.krzyzanowski@umlub.pl (A.K.)

**Keywords:** mesenchymal stem cells, perinatal stem cells, human breast milk, gestational hypertension, maternal hypothyroidism, microRNA-155, DOHaD paradigm

## Abstract

Perinatal mesenchymal stem cells (MSCs) are characterized by high phenotypic plasticity, allowing them to dynamically adapt to microenvironmental stimuli. However, the extent to which maternal pathophysiology and clinical history in vivo permanently alter their biological properties remains poorly understood. The aim of this study is to determine the influence of the maternal in vivo environment—including vascular stress (gestational hypertension), metabolic stress (hypothyroidism), and obstetric history (miscarriages, stillbirths)—on the transcriptomic, proteomic, and epigenetic profiles of growth factors in umbilical cord blood stem cells (UC-MSCs) and human milk stem cells (MM-MSCs). UC-MSCs (n = 51) and MM-MSCs (n = 78) were isolated from eligible donors. Global transcriptomic pathways associated with maternal hypertension were examined in UC-MSCs using Affymetrix expression microarrays. Targeted polymerase chain reaction (qPCR) validation was performed in both cell sources for key growth factors and receptors and for microRNA-155 in MM-MSCs. Paracrine protein concentrations (HGF, IGF-1, VEGF) in whole milk were quantified using ELISA. Microarray screening demonstrated that maternal hypertension is associated with a transcriptomic shift in the UC-MSCs towards a profibrotic signature, marked by an increased expression of the canonical Wnt signaling pathway and structural components of the extracellular matrix, while simultaneously suppressing the expression of genes associated with cell migration networks. qPCR confirmed that maternal hypertension is associated with significantly reduced *FGFR1* expression and a downward trend in *EGF* and *HGF* in UC-MSCs, while correlating with an asymmetric response in MM-MSCs. Maternal hypothyroidism was linked to a transcriptomic profile suggestive of a rescue response (a trend toward increased *IGF1* and *EGF* expression) in UC-MSCs, in contrast to a potential paracrine translation deficit (trends toward reduced VEGF, HGF, and IGF-1 protein levels) in the milk matrix. Importantly, a history of miscarriage correlated with alterations in the cellular mechanism of MM-MSCs (a trend toward increased *IGF1* and *EGF* mRNA expression), while previous stillbirths were associated with a systemic, threefold increase in free IGF-1 protein concentration in whole milk. This paracrine dynamic strongly correlated with miR-155 expression, suggesting a potential regulatory role in the lactation niche. Our findings suggest that the molecular and epigenetic makeup of perinatal stem cell niches is dynamically and persistently associated with the mother’s somatic health and reproductive history. This tissue-specific plasticity provides robust molecular support for the DOHaD paradigm, indicating that offspring stem cell profiles may directly reflect the maternal inheritance in vivo.

## 1. Introduction

Mesenchymal stem cells (MSCs) are multipotent, fibroblast-like cells with immunomodulatory and regenerative properties that are capable of differentiating into various mesenchymal cell lines [1,2,3,4]. Owing to their ability to influence surrounding tissues, MSCs have attracted considerable interest in the field of regenerative medicine [2]. Depending on the source—which may include bone marrow, adipose tissue, synovial membrane, placenta, liver, heart, umbilical cord, and umbilical cord blood—MSCs lines are characterized by diverse proliferative, secretory, migratory, and differentiation capabilities, as well as angiogenic potential and clonogenicity. They have also been shown to differ in gene expression profiles and membrane marker expression [1,5]. MSCs derived from dental tissues exhibit high proliferative capacity, whereas adipose tissue-derived MSCs and gingival MSCs are characterized by greater clonogenic potential [5].

Not only the tissue from which the cells were derived is important for the biological heterogeneity of MSCs, but also the extracellular environment, which consists of cytokines, proteases, neighboring cells, hormones, growth factors, and other signaling components [1]. Moreover, alterations in the cell environment, such as those associated with aging, tumors, autoimmune diseases, or hypoxia, are reflected in the molecular pathways and functional behavior of MSCs [1,6].

With advancing age, MSCs exhibit reduced proliferative potential, decreased viability, and impaired differentiation capacity [3]. Some MSCs obtained from the older group become senescent, which is characterized by irreversible cell cycle arrest, resistance to apoptosis, and metabolic dysfunction with molecular damage [7]. Interestingly, the gender of the donor has also been shown to influence the biological properties of MSCs [8].

Patients with type 2 diabetes and obesity exhibit a chronic inflammatory environment that adversely affects the immunomodulatory properties of MSCs, impairs their adipogenic potential through altered expression of *PPARγ* and *FABP4*, and reduces their differentiation capacity while promoting fibrosis [9]. A specific and critical example of stem cell phenotype modification by external factors is the intrauterine environment [10]. This phenomenon closely aligns with the DOHaD (Developmental Origins of Health and Disease) concept, which posits that the mother’s health and metabolic and hormonal homeostasis during pregnancy exert long-lasting epigenetic and transcriptomic influences on the offspring’s cells. Our previous studies demonstrated that the mother’s biochemical profile and perinatal factors are key regulators of fetal programming, determining the expression of paracrine genes (including *VEGF* and *TSG-6*) in umbilical cord cells, which directly modifies their initial proangiogenic and anti-inflammatory properties [10,11,12]. The biological properties of MSCs are further influenced by in vitro preconditioning (priming) [13,14].

In recent years, perinatal MSCs, including those derived from maternal milk (MM-MSCs) and umbilical cord tissue (UC-MSCs), have been gaining interest [2,15].

To comprehensively assess the functional reprogramming of perinatal stem cells, a targeted panel of ten key genes was selected based on their key roles in regulating pluripotency/stemness properties, paracrine activity, and cellular adaptation of MSCs. These markers can be functionally divided into: somatotropic and metabolic mediators (IGF1) [16,17,18,19], which regulate energy homeostasis, protein synthesis, and anti-apoptotic pathways; mitogenic and metabolic regulators (EGF, FGF2, FGFR1, FGFR3) [16,20,21,22,23,24], which drive cell survival and proliferation; angiogenic factors (HGF, PGF) [19,25,26,27,28], critical for vascular organization; neurotrophic and neuroprotective factors (GDNF, NGF) [17,29,30,31], which mediate tissue rescue and nervous system support; and markers of cellular plasticity (NES) [32,33,34], which reflect the undifferentiated progenitor status of cells. Assessment of this specific, interconnected network allows for the precise determination of how maternal clinical conditions reprogram the therapeutic and physiological potential of both UC-MSCs and MM-MSCs.

In a previous screening study, we demonstrated that maternal endocrine disorders, including diabetes and hypothyroidism, induce significant transcriptomic alterations in UC-MSCs, particularly affecting the WNT/PCP signaling pathway and extracellular matrix (ECM) remodeling [10]. The presented study is a direct response to the limitations of our previous pilot study from 2026 [10], in which we clearly indicated the need for experimental verification of initial screening results using quantitative methods in a much larger cohort of patients. In this work, we not only address this need through quantitative validation (qPCR) of targeted mitogenic axes but also expand the existing methodological framework. We introduce another common perinatal pathology–gestational hypertension—into the analysis. Crucially for a comprehensive understanding of the defense mechanisms, we for the first time compare the molecular plasticity and protein secretion (ELISA) of fetal-derived cells (UC-MSCs) with the lactation niches of the mother herself (MM-MSCs). Based on these findings, the present study aims to validate these pathway-specific gene expression changes in a larger cohort, extend the analysis to maternal hypertension, and compare the molecular reactivity of UC-MSCs with MM-MSCs.

## 2. Results

### 2.1. Demographic Characteristics of the Study Population

Ultimately, 129 participants were included in the analysis. Umbilical cord samples were collected from 51 participants, and human milk samples were collected from 78 participants. Demographic characteristics, anthropometric parameters, and clinical profiles of mothers and newborns in the study groups are presented in Table 1.

To ensure comparability between groups and to account for potential confounding factors, clinical variables were described in detail. The mean age of patients from whom umbilical cords were collected (UC-MSC group) was 33 years (age range: 21–46 years). In this group, 44 patients delivered by cesarean section (86%), 7 by vaginal delivery (14%), and 14 patients had a history of previous miscarriages. The mean age of patients from whom milk was collected (MM-MSC group) was 30.5 years (age range: 18–44 years). In this group, 48 patients (62%) delivered by cesarean section, 30 patients (38%) delivered by vaginal delivery, 6 patients had a history of previous stillbirths, and 22 patients had a history of previous miscarriages.

Critical to the homogeneity of the study cohort was the fact that all participants were nonsmokers. Patients diagnosed with gestational hypertension were treated with methyldopa, while patients with hypothyroidism received standard levothyroxine. Regarding comorbidities, in the MM-MSC group, three patients were diagnosed with diabetes, including two with concurrent hypothyroidism and one with hypertension. In the UC-MSC group, diabetes was a comorbidity in six patients, specifically two with hypertension and four with hypothyroidism.

To carefully account for these potential confounders, relevant clinical variables were incorporated into multivariate general linear models (GLMs). For the lactation cohort (MM-MSC), models were adjusted for maternal pre-pregnancy body mass index (BMI), mode of delivery, and maternal diabetes. For the umbilical cord cohort (UC-MSC), where complete pre-pregnancy BMI data were not available for all participants, models were appropriately adjusted for alternative key perinatal variables: gestational age, neonatal birth weight, mode of delivery, and maternal diabetes.

### 2.2. Confirmation of the Phenotype and Purity of Isolated MSCs Populations

**Figure 1:**
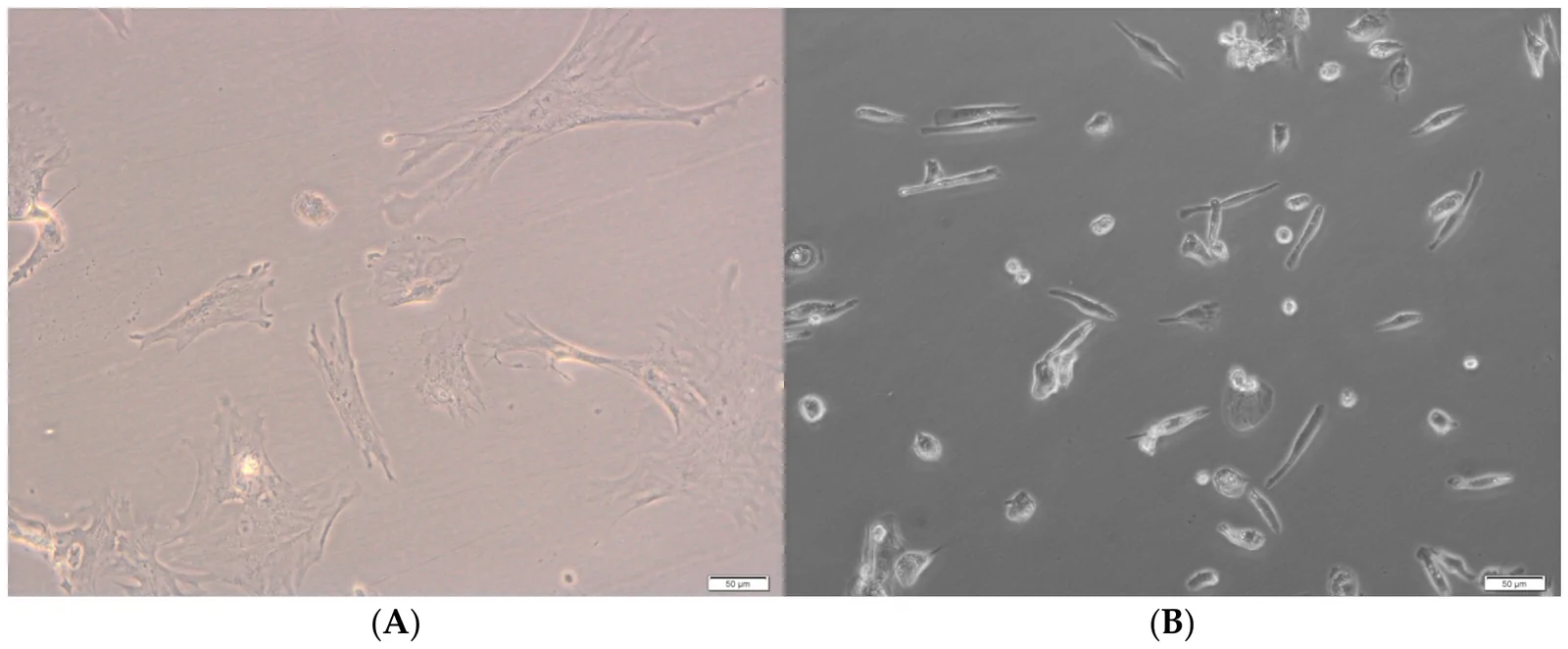
Morphological characterization of perinatal MSCs in initial culture. Microscopic images were recorded in brightfield (phase contrast) using an Olympus CKX41 inverted microscope equipped with an Olympus XC50 digital camera. Scale bar: 50 µm. (**A**) UC-MSCs: adherent, strongly flattened cells with a classic, spindle-shaped (fibroblast-like) morphology visible. The cells exhibit clear structural boundaries and long, branching cytoplasmic processes, typical of the early stage of adhesion and colonization on a plastic substrate (200× magnification). (**B**) MM-MSCs: adherent cells exhibiting phenotypic heterogeneity typical of the early stage of stem cell isolation from biological fluids visible. In addition to the dominant spindle-shaped (fibroblast-like) cells with distinct cytoplasmic projections, single cells of polygonal and rounded shape are visible, attached to the plastic substrate (100× magnification).

Prior to molecular analyses, the identity and purity of the cell populations isolated from both umbilical cord (UC-MSCs) and human milk (MM-MSCs) were verified according to the commonly accepted criteria of the International Society for Cell and Gene Therapy (ISCT). Brightfield phase contrast microscopic analysis confirmed the ability of both cell types to adhere to the plastic substrate and revealed a characteristic, fibroblast-like morphology (Figure 1A,B).

Additionally, to confirm the antigenic profile, randomly selected samples from both niches were subjected to flow cytometric analysis (including assessment of the expression of CD73, CD90, and CD105 markers, while simultaneously lacking hematopoietic markers). Because the procedure for isolating and culturing MSCs is routine in our laboratory and has been fully validated, detailed flow cytometric evaluation was not performed individually for each sample (example histograms from the analysis are presented in the Supplementary Materials—Supplementary Figure S1). The obtained morphological and phenotypic profiles unequivocally confirmed that the tested material constituted pure populations of MSCs.

### 2.3. Qualitative Baseline and Tissue-Specific Signatures

In the first stage of the analysis, the qualitative baseline expression profile of a panel of 10 selected genes (*EGF*, *FGF2*, *FGFR1*, *FGFR3*, *GDNF*, *HGF*, *IGF1*, *NES*, *NGF*, and *PGF*) was assessed under control conditions for both perinatal cell niches. qPCR revealed distinct tissue-specific differences in the presence of transcription. UC-MSCs demonstrated stable, constitutive expression of all 10 genes across all biochemical replicates. In contrast, MM-MSCs demonstrated a restricted profile—transcripts for neurotrophic factors *GDNF* and *NGF* and angiogenic factor *PGF* were completely undetectable, regardless of the mother’s health status or obstetric history.

**Figure 2:**
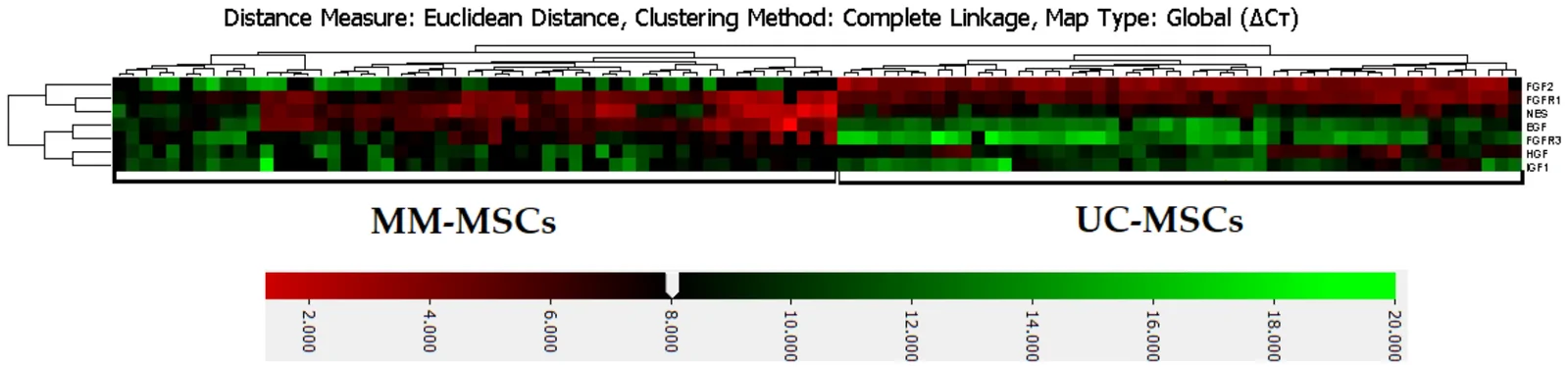
Global expression profile of the studied genes in MM-MSCs and UC-MSCs. Heatmap combined with two-way hierarchical clustering analysis (rows and columns). The data matrix is based on global ΔCT (Global DeltaCT) values obtained in the qPCR study. Euclidean distance was used as a measure of similarity (distance), and cluster agglomeration was performed using the Complete Linkage method. The color scale bar at the bottom reflects ΔCT values: Red (low values, close to 2000) indicates a low cycle detection threshold, corresponding to a high level of transcriptional expression. Black (middle value 8000) indicates a moderate level of expression. Green (high values, up to 20,000) indicates a high cycle detection threshold, corresponding to a low level of or no expression. The vertical dendrogram (left) illustrates the similarity of the expression profiles of the evaluated genes *(FGF2*, *FGFR1*, *NES*, *EGF*, *FGFR3*, *HGF*, *IGF1*). The horizontal dendrogram (top) shows the phenotypic division of the samples, clearly separating the MM-MSCs cohort from the UC-MSCs cohort into two main clusters. Generated using ExpressionSuite Software (Life Technologies).

**Figure 3:**
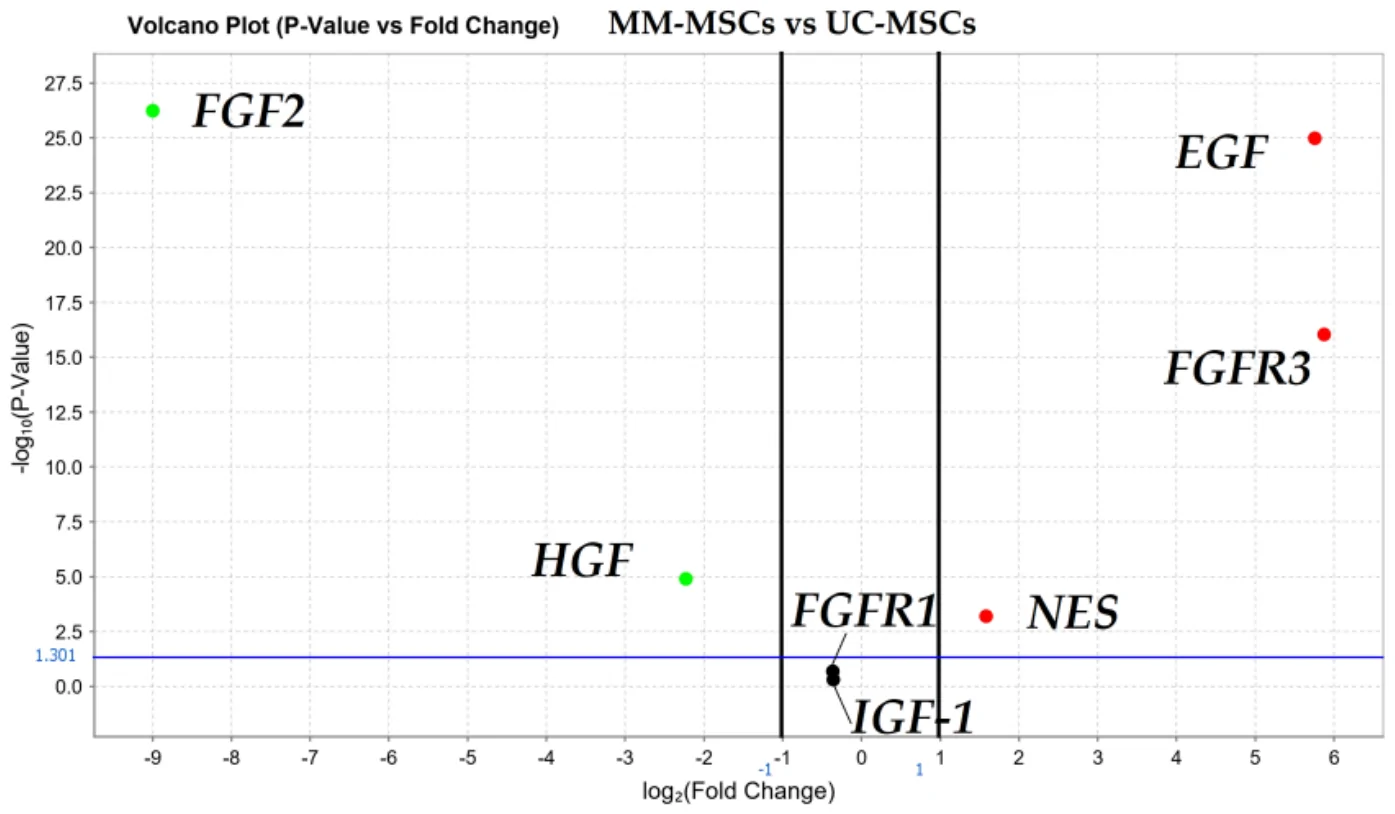
Volcano plot comparing gene expression profiles between MM-MSCs and UC-MSCs. The horizontal axis represents the fold change in expression expressed as log_2 fold change, and the vertical axis represents statistical significance as −log_10 (*p*-value). The horizontal blue line indicates the threshold of statistical significance (*p* = 0.05; −log_10P = 1.301). Vertical black lines define the boundaries of significant change in expression level (fold change = ±2, corresponding to log_2FC = ±1). Red points (right): genes with significantly higher expression in milk stem cells (*EGF*, *FGFR3*, *NES).* Green points (left): genes with significantly higher expression in umbilical cord cells (*FGF2*, *HGF*). Black points: transcripts with no significant differences in expression (*FGFR1*, *IGF-1*). The graph was generated using ExpressionSuite Software (Life Technologies).

To identify differences in the underlying molecular profile of the studied cell niches, raw qPCR data were subjected to comparative analysis (MM-MSCs vs. UC-MSCs) using ExpressionSuite Software (v1.3, Life Technologies, Carlsbad, CA, USA) (Figure 2 and Figure 3). The analysis revealed a clear transcriptomic separation between the two stem cell sources. MM-MSCs were characterized by strong overexpression of *EGF* (log_2FC ≈ 5.8; −log_10P ≈ 25) and *FGFR3* (log_2FC ≈ 5.9; −log_10P ≈ 16). This cohort also showed significantly higher levels of *NES* expression (log_2FC ≈ 1.5).

In contrast, UC-MSCs demonstrated dramatically higher *FGF2* expression (log_2FC ≈ −9; −log_10P ≈ 26), representing a more than 500-fold difference on a linear scale. Furthermore, significantly higher *HGF* levels were found in the UC-MSC group (log_2FC ≈ −2.2; −log_10P ≈ 5). Transcripts for the *FGFR1* and *IGF-1* genes were below the accepted cutoff thresholds, showing no statistically significant differences between the studied perinatal niches.

### 2.4. Maternal Hypertension Is Associated with a Fibrotic and Suppressive Transcriptomic Profile Phenotype in UC-MSCs

#### 2.4.1. Microarray Analysis

**Figure 4:**
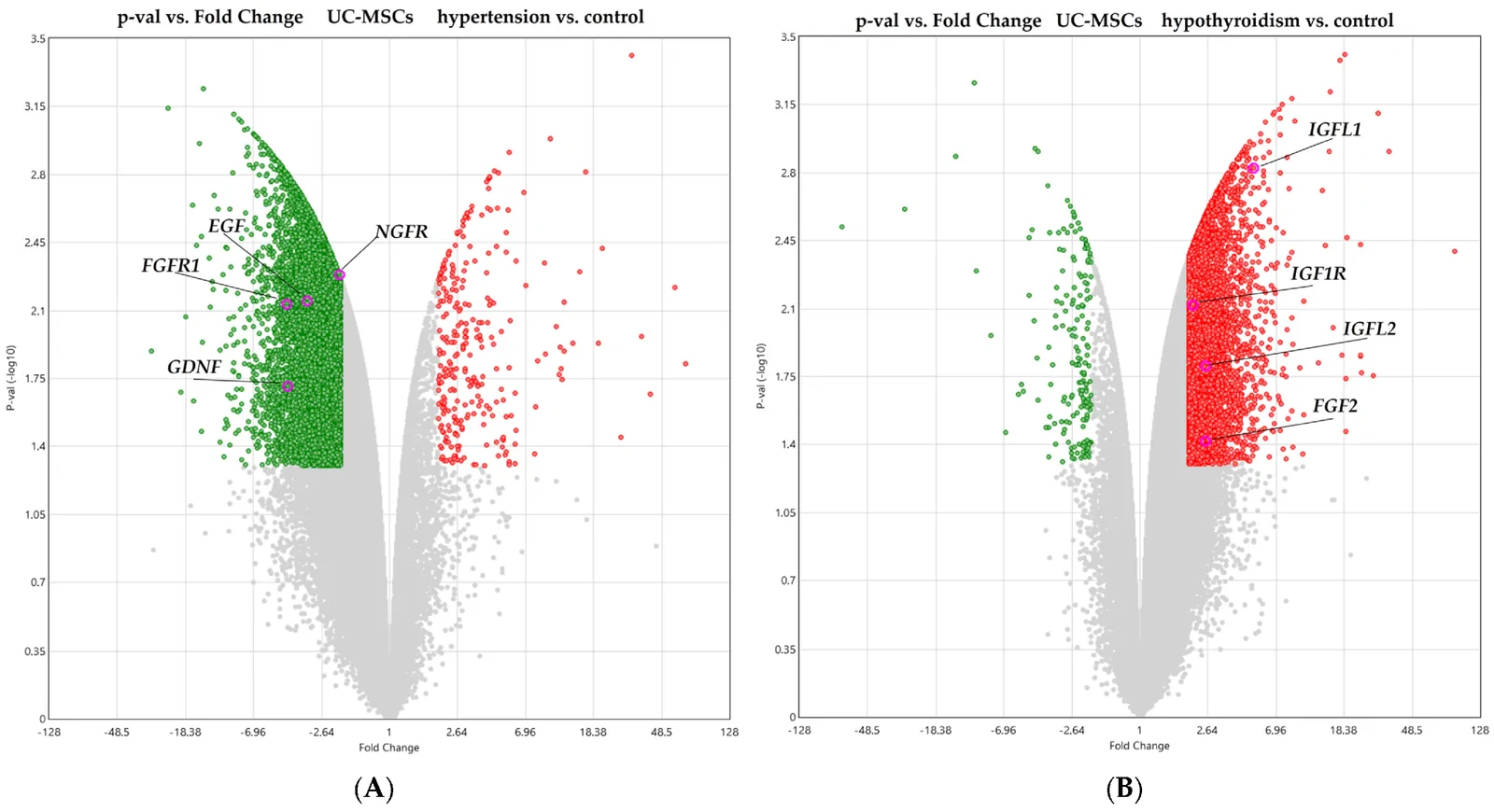
(**A**). Gene expression profile of UC-MSCs isolated from patients with gestational hypertension compared to the control group. Volcano plot generated using Transcriptome Analysis Console (TAC) Software (v4.0.2, Affymetrix/Thermo Fisher Scientific, Santa Clara, CA, USA). The horizontal axis represents fold change (fold change on a bilinear scale, where negative values indicate decreased expression). The vertical axis represents statistical significance expressed as −log_10 (*p*-value). Green points (left): genes with significantly decreased expression in the hypertension group (fold change ≤ −2, *p* < 0.05). Red points (right): genes with significantly increased expression in the hypertension group (fold change ≥ 2, *p* < 0.05). Gray points: transcripts not meeting the criteria for statistical significance or fold change. Purple circles: selected biologically relevant growth factors and neurotrophic factors (*NGFR, EGF, FGFR1, GDNF*) showing strong transcriptional suppression under maternal hypertension. Gene expression profile of UC-MSCs isolated from patients with gestational hypertension (n = 2) compared to the control group (n = 2). (**B**). Gene expression profile of UC-MSCs isolated from patients with hypothyroidism compared to the control group. Volcano plot generated using Transcriptome Analysis Console (TAC) Software (Affymetrix/Thermo Fisher Scientific). The horizontal axis represents the fold change in expression (fold change on a bilinear scale, where positive values indicate increased expression). The vertical axis represents statistical significance expressed as −log_10 (*p*-value). Red points (right): genes with significantly increased expression in the hypothyroid group (fold change ≥ 2, *p* < 0.05). The characteristic high density of points in this zone illustrates global activation of the cellular transcriptome. Green points (left): genes with significantly decreased expression in the hypothyroid group (fold change ≤ −2, *p* < 0.05). Gray dots: transcripts not meeting statistical significance or fold change criteria. Purple circles: genes identified in the global analysis as key components of the growth factor signaling axis, showing strong overexpression: *IGFL1*, *IGF1R*, *IGFL2*, and *FGF2.* Gene expression profile of UC-MSCs isolated from patients with hypothyroidism (n = 3) compared to the control group (n = 2).

Global transcriptomic analysis performed with TAC software revealed a profound reprogramming of the molecular profile of UC-MSCs under the influence of maternal gestational hypertension (Figure 4A). Data visualization as a volcano plot revealed a striking asymmetry in gene regulation, dominated by massive global transcriptional silencing.

Within the cluster of downregulated genes, key regulators responsible for the trophic, mitogenic, and neuroprotective potential of stem cells were identified. Maternal hypertension induced a significant suppression of *EGF* (FC ≈ −3.5; −log_10P > 2.1) and *FGFR1* (FC ≈ −4.5; −log_10P ≈ 2.1). Furthermore, analyses revealed a strong silencing of the neurotrophic axis, manifested by a significant decrease in the expression of *GDNF* (FC ≈ −4.5; −log_10P ≈ 1.7) and *NGFR* (FC ≈ −2.0; −log_10P ≈ 2.3).

Exploratory microarray profiling revealed that maternal hypertension substantially alters the functional landscape of fetal MSCs. Functional enrichment analysis of upregulated transcripts revealed a clear shift towards structural modification of the extracellular matrix (ECM) and tissue remodeling. The most statistically significant enrichment was observed for “collagen-containing extracellular matrix” (GO:0062023, *p*_adj = 1.204 × 10^−9^) and “regulation of cell migration” (GO:0030334, *p*_adj = 1.410 × 10^−7^). Furthermore, increased expression of genes involved in “extracellular matrix organization” (GO:0030198, *p*_adj = 8.273 × 10^−4^) and “positive regulation of canonical Wnt signaling” (GO:0090263, *p*_adj = 1.308 × 10^−2^), a classical pathway involved in tissue fibrogenesis, was observed in hypertensive MSCs (Table 2). These initial findings from the global analysis served as the basis for targeted qPCR validation in an expanded patient cohort.

In contrast, genes whose expression was downregulated under maternal hypertension were highly enriched in their regulation of basic transcriptional and physiological mechanisms. Hypertensive MSCs showed profound suppression of “DNA-binding transcription factor activity” (GO:0003700, *p*_adj = 1.063 × 10^−7^) and “regulation of transcription by RNA polymerase II” (GO:0006357, *p*_adj = 6.493 × 10^−4^). Furthermore, important stem cell functional properties, such as “cell migration” (GO:0016477, *p*_adj = 4.551 × 10^−3^) and “transmembrane transport” (GO:0055085, *p*_adj = 1.873 × 10^−3^), were significantly reduced. Importantly, significant suppression was identified for the “negative regulation of blood pressure” pathway (GO:0045776, *p*_adj = 8.927 × 10^−3^) (Table 3).

Selected pathways of key biological importance, directly related to the pathophysiology of vascular stress, are highlighted in shaded areas, including processes responsible for extracellular matrix (ECM) organization, collagen synthesis, cell migration, and activation of the canonical Wnt signaling pathway. Abbreviations: GO:BP—Biological Process; GO:CC—Cellular Component; GO:MF—Molecular Function; *p*_adj—adjusted *p*-value.

#### 2.4.2. qPCR Validation

To confirm these results, qPCR validation was performed on a larger cohort, targeting key regulators of these processes. Consistent with the transcriptomic suppression observed in microarrays, the expression of *FGFR1* was significantly reduced in UC-MSCs exposed to maternal hypertension after applying the strict multiple testing correction (*p* = 0.006). A strong downward trend was also observed for *EGF* and *HGF* expression, though these did not reach the conservative formal significance threshold of *p* < 0.01 (Table 4). Expression of the remaining seven genes examined, including *GDNF* and *NGF*, did not differ significantly between the hypertensive and control groups.

### 2.5. Maternal Hypothyroidism Is Linked to Alterations in the UC-MSCs Transcriptome

#### 2.5.1. Microarray Analysis

The global transcriptomic profile obtained using TAC revealed a massive, asymmetric response of the UC-MSC genetic apparatus under maternal hypothyroidism (Figure 4B). Data visualization in a volcano plot clearly confirmed the powerful predominance of gene activation over gene silencing, as evidenced by the dense accumulation of transcripts in the overexpression zone. The analysis focused on genes involved in cellular trophic and mitogenic pathways. TAC software identified a coordinated, statistically significant upregulation of key components of the insulin-like growth factor (IGF) axis. A strong increase in gene expression of *IGFL1* (FC ≈ 5.0; −log_10P ≈ 2.8), *IGFL2* (FC ≈ 2.6; −log_10P ≈ 1.8) and the receptor for this pathway—*IGF1R* (FC ≈ 2.3; −log_10P ≈ 2.1) was observed. Furthermore, preliminary microarray analysis indicated a significant overexpression of *FGF2* (FC ≈ 2.5; −log_10P ≈ 1.4).

Microarray analysis revealed profound transcriptome remodeling in MSCs under hypothyroidism, characterized by striking asymmetry in gene regulation. Of the 4009 significantly altered transcripts (*p* < 0.05), the vast majority (3900 genes) were upregulated (FC > 2), whereas only 85 genes were downregulated (FC < −2). Functional enrichment of the cohort of downregulated genes revealed highly specific changes focused on membrane homeostasis and molecular trafficking. Importantly, the ABC transporter pathway (KEGG:02010) was significantly enriched (*p* = 0.033) (Table 5). Furthermore, a significant enrichment of the G protein complex (CORUM:1615, *p* = 1.77 × 10^−4^) was found.

In contrast, a large cohort of upregulated genes showed widespread activation of cellular processes. The most important biologically relevant GO:BP categories were primarily related to metabolic and signaling control, including “regulation of primary metabolic process” (GO:0080090, *p*_adj = 2.06 × 10^−19^) and “intracellular signal transduction” (GO:0035556, *p*_adj = 2.24 × 10^−14^). Furthermore, hypothyroid MSCs showed robust activation of stress adaptation programs, as evidenced by enrichment in the “stress response” (GO:0006950, *p*_adj = 4.55 × 10^−8^) and the “stress-induced protein mRNA and metabolite” WikiPathways “pathway” (WP3953). Analysis of cellular components confirmed that these transcriptionally active products were widely distributed in the cytoplasm and nucleoplasm, indicating global intracellular and nuclear response networks (Table 6).

#### 2.5.2. qPCR Validation

The reproducible activation of *IGF* family receptors and ligands identified in the TAC program served as a direct premise for targeted molecular validation (qPCR) of the entire signaling axis in an expanded cohort of patients. The qPCR study indicated an observable upward trend in both *IGF1* and *EGF* expression in UC-MSCs from patients with hypothyroidism compared to controls (Table 7). However, following the application of the Bonferroni correction, these elevated levels did not cross the adjusted threshold for statistical significance (*p* < 0.01). The expression of the remaining eight genes examined in UC-MSCs did not show significant differences depending on the presence of hypothyroidism.

Data are presented as the mean log-transformed value of relative expression (log_10RQ ± SD). Statistical significance between the hypothyroidism group and the control group (physiological pregnancies) was assessed using the parametric Student’s *t*-test for independent samples.

### 2.6. Multifactorial Analysis of Gene Expression in UC-MSCs

To determine whether the observed transcriptomic alterations in UC-MSCs were independently associated with maternal pathologies rather than confounded by clinical variables, general linear models (GLMs) were constructed. The models were adjusted for gestational age, neonatal birth weight, mode of delivery, and maternal diabetes, and both statistical significance and effect size (partial eta squared, ηp^2^) were evaluated.

In the cohort of mothers with hypertension, multivariable analysis confirmed that maternal hypertension was a strong independent predictor of reduced gene expression. The downregulation of *FGFR1* (F(1, 43) = 11.54, *p* = 0.001) was associated with a particularly large effect size (ηp^2^ = 0.212), indicating that maternal hypertension alone accounted for more than 21% of the variance in *FGFR1* expression. Likewise, the reduced expression of *HGF* (F(1, 42) = 6.85, *p* = 0.012, ηp^2^ = 0.140) and *EGF* (F(1, 41) = 4.75, *p* = 0.035, ηp^2^ = 0.104) remained statistically significant after adjustment for all covariates. Interestingly, mode of delivery also exerted an independent effect on *EGF* expression (*p* = 0.021), whereas birth weight, gestational age, and maternal diabetes were not significant confounding factors in any of the hypertension models (Table 8).

In the cohort of mothers with hypothyroidism, the GLM demonstrated that the increased *EGF* expression was independently associated with maternal thyroid status (F(2, 40) = 3.52, *p* = 0.039, ηp^2^ = 0.150), irrespective of the included clinical covariates. In contrast, the previously observed association with *IGF1* expression was no longer statistically significant after multivariable adjustment (F(1, 42) = 1.87, *p* = 0.179). Importantly, none of the covariates (gestational age, birth weight, mode of delivery, or maternal diabetes) reached statistical significance in the IGF1 model (all *p* > 0.40) (Table 8). These findings suggest that the attenuation of the initial association is more likely attributable to the reduced statistical power inherent to multivariable modeling within a relatively small subgroup than to a true confounding effect of the evaluated clinical variables.

### 2.7. The Impact of Maternal Conditions on Breast Milk MSCs and Secretory Profiles

Analysis of the gene expression profile in MM-MSCs was performed on samples obtained from 54 patients whose RNA isolation efficiency met the methodological criteria. The protein concentration was assessed using ELISA in whole milk from 78 patients.

Based on the qPCR analysis, the expression of the *NES* gene in MM-MSCs was statistically significantly higher in the group of patients with hypertension compared to the control group (*p* = 0.003). Additionally, trends toward higher expression of *EGF* and *FGFR3*, and lower expression of HGF and IGF1 were observed, although these changes were not statistically significant under the adjusted *p* < 0.01 threshold. The remaining genes examined did not show significant differences in expression depending on the presence of hypertension in MM-MSCs. HGF, IGF, and VEGF proteins tended to be lower in patients with hypertension, but these results were not statistically significant (Table 9).

Taking into account the analysis of the expression of the studied genes in MM-MSCs depending on the presence of hypothyroidism, trends toward increased expression of the *EGF* gene and decreased expression of the *HGF* and *IGF1* genes were observed compared to the control group, but they lacked formal significance after correction. A similar pattern emerged in whole milk protein concentrations: VEGF levels showed a visible reduction in patients with hypothyroidism, while HGF and IGF levels also tended to be lower, but none reached the strict statistical significance threshold (Table 10).

### 2.8. Obstetric History as a Predictor of Milk Trophic Value

The final stage of the analysis assessed whether the patients’ difficult obstetric history—including miscarriages and stillbirths—had a long-term impact on the transcriptional profile of isolated stem cells (MM-MSCs) and the final protein composition of whole milk.

Quantitative qPCR analysis revealed trends toward increased *EGF*, *IGF1*, and *FGFR1* gene expression in the MM-MSC population in women with a history of miscarriages compared to those without such complications, though these did not reach the rigorous significance threshold (Table 11). Similarly, we noted a trend suggesting higher IGF and HGF protein concentrations in the milk of patients with a history of stillbirths (Table 11).

### 2.9. Multivariable Analysis of the Lactational Niche (MM-MSCs and Whole Milk)

To confirm the independent effects of maternal conditions on the lactational microenvironment, GLMs were constructed adjusting for pre-pregnancy BMI, mode of delivery, and maternal diabetes.

In the cohort of mothers with hypertension, multivariable analysis demonstrated that maternal hypertension was a strong independent predictor of trophic factor activation in MM-MSCs. Specifically, maternal hypertension independently increased the expression of *HGF* (F(1, 26) = 4.60, *p* = 0.041, ηp^2^ = 0.150), *NES* (F(1, 47) = 8.22, *p* = 0.006, ηp^2^ = 0.149), *FGFR3* (*p* = 0.031), and *EGF* (*p* = 0.048) (Table 12). None of the evaluated covariates (pre-pregnancy BMI, mode of delivery, or maternal diabetes) exerted significant confounding effects, indicating that the transcriptomic activation observed in MM-MSCs was independently associated with maternal hypertension. As expected, given the limited sample size, the initially significant univariate association for IGF1 was no longer statistically significant after multivariable adjustment (*p* = 0.191).

A striking independent secretory signature was also identified in relation to maternal obstetric history. A history of stillbirth emerged as a strong independent predictor of increased concentrations of trophic proteins in whole human milk. After adjustment for potential metabolic confounders, a history of stillbirth remained significantly associated with elevated levels of HGF (F(1, 47) = 11.33, *p* = 0.002, ηp^2^ = 0.194), IGF (F(1, 45) = 10.35, *p* = 0.002, ηp^2^ = 0.187), and VEGF (*p* = 0.033) (Table 12). The large effect sizes observed for HGF and IGF, each explaining nearly 20% of the variance, suggest substantial and persistent alterations in the paracrine secretory profile of the mammary gland associated with previous stillbirth.

In contrast, in the multivariable models evaluating maternal hypothyroidism and a history of miscarriage, the previously observed univariate associations (e.g., involving IGF1 and EGF) did not retain statistical significance (all *p* > 0.05). Importantly, none of the included covariates reached statistical significance in these models, indicating that the attenuation of these associations is more likely attributable to the reduced statistical power associated with analyses of relatively small subgroups rather than to confounding by the evaluated clinical variables. Therefore, these molecular alterations should be interpreted cautiously as hypothesis-generating observations requiring confirmation in larger cohorts.

### 2.10. Correlation Matrix Between miR-155 and Growth Factor Profiles in MM-MSCs

**Figure 5:**
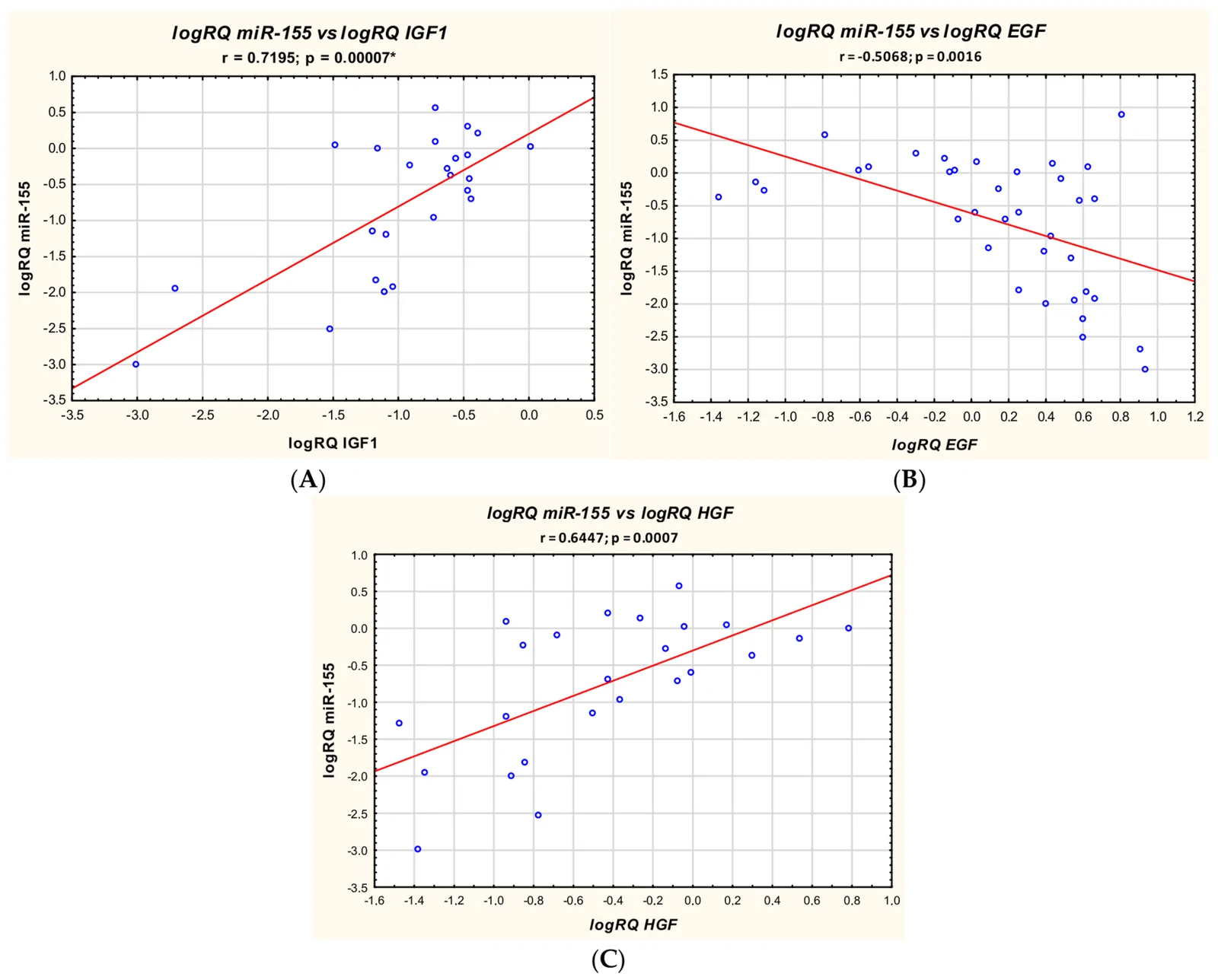
Scatterplot (**A**). Gene expression (log10RQ) of *IGF1* vs. miR-155 expression (log10RQ) (**B**). Gene expression (log10RQ) of *EGF* vs. miR-155 expression (log10RQ). (**C**). Gene expression (log10RQ) of *HGF* vs. miR-155 expression (log10RQ). Spearman correlation (* miR-155 expression was not available for the entire cohort of 54 MM-MSC samples).

To assess the potential epigenetic regulation governing the paracrine profile of human milk stem cells, correlation analysis was performed between miR-155 expression–previously profiled in this patient cohort [35] and transcripts of target growth factors. In MM-MSCs, miR-155 expression showed a strong and statistically significant positive correlation with both *IGF1* (r = 0.72, *p* < 0.05) and *HGF* (r = 0.64, *p* < 0.05). In contrast, a statistically significant negative correlation was found between miR-155 levels and *EGF* expression (r = −0.51, *p* < 0.05) (Figure 5A–C).

### 2.11. Summary of Results

To facilitate comprehensive interpretation of the obtained data and integrate results from different levels of molecular validation, this section summarizes key transcriptomic and proteomic changes. Multilevel analysis—including global microarray screening (TAC/g:Profiler), targeted mRNA validation (qPCR), and assessment of paracrine secretion (ELISA—milk)—revealed that UC-MSCs and MM-MSCs demonstrate distinct, highly specific adaptation strategies to the pathological maternal microenvironment (Table 13).

Based on the integrated matrix of results, four overarching biological conclusions can be drawn, providing a direct starting point for a discussion of the mechanisms of fetal programming:

Maternal hypertension is associated with a state in which a decrease in *FGFR1* and a downward trend in *EGF* and *HGF* are observed at the transcript level in UC-MSCs, while MM-MSCs exhibit a trend toward higher expression of their default trophic shield: *EGF*, *FGFR3*, *IGF1*, and *NES*.

In hypothyroidism, we observe completely different responses. The UC-MSCs profile suggests an upregulation of the receptor-ligand axis of the insulin-like growth factor system (*IGFL1*, *IGFL2*, *IGF1R*, *IGF1*). In MM-MSCs, on the other hand, a decrease in IGF1 occurs at both the mRNA and protein levels.

Comparison of qPCR and ELISA results in the milk of patients with hypothyroidism revealed the presence of a metabolic block. MM-MSCs show transcriptional mobilization only of *EGF* mRNA, while the *IGF1* and *HGF* genes are downregulated, and we also observe a decrease in protein concentrations (VEGF, HGF, IGF-1) in whole milk.

A maternal history of miscarriage and stillbirth may be associated with a lasting molecular imprint for subsequent lactations. This potential memory operates at two levels: past miscarriages correlate with an altered cellular transcriptional machinery of MM-MSCs (increasing *IGF1* and *EGF* mRNA expression), while a history of stillbirth causes a systemic increase in free IGF-1 protein secretion in whole milk. The correlation patterns observed between miR-155 and target growth factors suggest that miR-155 may play a regulatory role in the epigenetic tuning of the lactation niche.

## 3. Discussion

### 3.1. Comparison of Growth Factor Expression in UC-MSCs and MM-MSCs

Understanding how maternal pathologies modify the phenotype of MSCs first requires defining their initial transcriptional identity. This study represents, to the best of our knowledge, the first direct molecular comparison of UC-MSCs and MM-MSCs. The baseline differential profile we obtained clearly indicates that these two perinatal niches, despite sharing classical mesenchymal features, possess distinctly different, tissue-specific functional arsenals.

MSCs are present in the majority of human tissues. Numerous studies have demonstrated that the molecular profile of MSCs is highly dependent on their tissue of origin [36,37,38]. This is largely attributable to the specific microenvironment of a given tissue, which modulates gene expression and consequently influences cellular biology. Depending on the source, MSCs exhibit distinct proangiogenic, immunomodulatory, and anti-apoptotic properties, as well as different differentiation potentials. For example, MSCs derived from umbilical cord blood possess greater osteogenic potential than UC-MSCs, whereas UC-MSCs display enhanced proangiogenic capacity [37]. Many similar comparisons have been reported in the literature. In the present study, we aimed to provide novel insights into the comparative biological potential of UC-MSCs and MM-MSCs.

We observed that UC-MSCs expressed all analyzed genes, in contrast to MM-MSCs, which lacked expression of the proangiogenic factor *PGF* as well as *GDNF* and *NGF*, both of which are involved in nervous system development. This finding is particularly relevant in the context of the neurogenic potential of these cells. *GDNF* and *NGF* are known to participate in neurogenesis and neuronal protection [39]. The expression of these genes by UC-MSCs suggests both a capacity for neuronal differentiation and a neuroprotective potential. Conversely, the absence of their expression in MM-MSCs may indicate a reduced neurogenic capacity.

Despite the lack of *NGF* and *GDNF* expression in MM-MSCs, differentiation into neural-like cells in vitro remains possible when appropriate culture conditions are applied [40]. This research paradox is further supported by our baseline observation of nestin (*NES*) expression. Nestin, a canonical intermediate filament protein and a recognized marker of neural progenitor cells and cellular plasticity, was highly expressed in MM-MSCs. The persistence of high levels of *NES*, despite the absence of mature neurotrophins such as *NGF* and *GDNF*, strongly suggests that MM-MSCs maintain a highly plastic, undifferentiated, primordial neuroectodermal state. This intrinsic molecular blueprint likely underlies their predisposed capacity for neuronal transdifferentiation upon specific microenvironmental cues in vitro, but further studies are required to confirm this.

However, from a functional perspective, the paracrine properties of MSCs are of greater importance. Under in vivo conditions, the secretome plays a pivotal role in mediating biological effects, and its composition depends on the positive expression of specific genes.

Numerous studies have demonstrated the supportive effects of UC-MSCs on neural cell function. Guo et al. reported that UC-MSCs promote the proliferation and viability of Schwann cells [41], while Chi et al. observed antioxidant and anti-apoptotic effects on dopaminergic neurons [42]. These findings suggest that UC-MSCs possess the ability to secrete a neurotrophic secretome, which was further confirmed by Guo et al. [41]. To the best of our knowledge, no studies have demonstrated a comparable effect for MM-MSCs. Consistent with these observations, our analysis did not detect *NGF* or *GDNF* in milk samples.

Furthermore, UC-MSCs exhibited higher expression levels of *HGF* and *FGF2*. This observation is also consistent with the findings of Guo et al., who reported elevated concentrations of these growth factors in conditioned media derived from UC-MSC cultures. The authors emphasized the important role of these factors in neurogenesis [41].

In contrast, MM-MSCs demonstrated increased expression of *EGF* and *FGFR3*. *EGF* has been repeatedly identified in human milk and is recognized for its anti-apoptotic, anti-inflammatory, and protective effects on the neonatal intestine [43]. Our findings suggest that MM-MSCs may contribute to the elevated EGF levels observed in breast milk, as these cells exhibit enhanced *EGF* expression. Therefore, the elevated *FGFR3* expression identified in our study represents a novel and groundbreaking finding in MM-MSC biology. Although the important role of the FGF/FGFR axis in stromal tissue homeostasis is widely recognized, the selective regulation of *FGFR3* expression in lactating stem cells suggests a specialized, tissue-specific sensitivity to local fibroblast growth factors in the mammary gland niche. This receptor predisposition potentially regulates intrinsic proliferation dynamics and cell-matrix interactions unique to the breast milk microenvironment, constituting a distinct physiological signature that requires further functional investigation.

Taken together, these basic characteristics reveal a fundamental functional dichotomy between the two sources of perinatally derived stem cells. UC-MSCs likely have an evolutionary vascular-stromal pattern, relying heavily on the HGF/FGF2 axis to support umbilical and placental structures. In contrast, MM-MSCs possess a protective epithelial shield characterized by high expression of *EGF*, *FGFR3*, and *NES*, likely adapted to support the neonatal gut and local niche plasticity. As discussed below, this fundamental functional polarity dictates how each niche undergoes molecular remodeling under maternal pathological stress.

It should be emphasized that the observed differences were detected at the molecular level. Although the findings suggest that maternal conditions may influence the functional properties of the investigated cells, changes in gene expression alone cannot be considered definitive evidence of altered cellular function. In the absence of dedicated functional assays, it is not possible to determine with certainty whether the observed differences in gene expression translate into biologically meaningful changes in the therapeutic or regenerative potential of these cells.

### 3.2. Effect of Maternal Hypertension on Gene Expression in UC-MSCs and MM-MSCs

We observed a statistically significant decrease in the expression of the genes *FGFR1, HGF,* and *EGF* in UC-MSCs derived from women with hypertension. The involvement of EGF in the pathomechanism of hypertension has been previously demonstrated. Activation of EGFR has been shown to induce angiotensin II, which, through the renin-angiotensin-aldosterone system, increases blood pressure. However, it has also been observed that the primary role of EGFR in hypertension is related to vascular remodeling. Moreover, aortic smooth muscle cells from hypertensive rats have been shown to be more sensitive to the proliferative effects of EGF than those from normotensive animals. Additionally, Lundstam et al. demonstrated a positive correlation between serum EGF levels and diastolic blood pressure [44,45,46,47,48].

While these literature findings generally suggest an increased systemic *EGF* expression or sensitivity in hypertensive individuals as a part of vascular remodeling, our results demonstrate the exact opposite relationship in perinatal stem cells. This is highly consistent with findings by Armant et al. in preeclampsia, where reduced EGF levels were observed in both the placenta and plasma of affected patients [49]. Therefore, it can be assumed that the hypertensive environment may inhibit UC-MSC proliferation and reduce their involvement in vascular remodeling. Therefore, reduced expression of *EGF* in the context of hypertension and PE is an unfavorable phenomenon and likely reflects impaired EGF signaling [21].

It should be emphasized that our investigations were limited to the molecular level. Therefore, the impact of the hypertensive environment on the biological functions of UC-MSCs warrants further confirmation in functional studies.

A similar mechanism appears to apply to HGF and FGFR1 [25,26,50]. Both growth factors are elevated in patients with hypertension, likely representing a compensatory mechanism aimed at protecting blood vessels from damage caused by chronically elevated blood pressure. However, our results indicate that, at the molecular level, this compensatory mechanism is not active in UC-MSCs derived from women with hypertension.

Our findings suggest that the hypertensive environment may impair the paracrine and autocrine activities of UC-MSCs, including their proliferative, proangiogenic, antifibrotic, and antiapoptotic capacities. Nevertheless, further studies are needed to confirm these effects at the protein level and through functional assays.

To corroborate these findings, an independent analysis was conducted in MM-MSCs and yielded comparable results. Specifically, reduced *HGF* and *IGF1* expression was detected in samples obtained from women with hypertension. Furthermore, analysis of protein concentrations in whole milk demonstrated lower levels of HGF and IGF1 in the hypertensive group, although these differences were not statistically significant.

IGF1 has been demonstrated to exert anti-apoptotic effects under hypertensive conditions [51]. In our study, reduced *IGF1* expression in MM-MSCs and decreased IGF1 levels in the milk of hypertensive mothers were observed. This finding may suggest impaired cell survival compared to cells not exposed to a hypertensive environment. This hypothesis should be further validated through functional assays evaluating apoptosis.

A number of studies have reported increased levels of growth factors in the context of hypertension, which are considered to play a protective role [52]. In contrast, our findings suggest that this mechanism is not engaged in UC-MSCs and MM-MSCs. An explanation for this observation may be provided by Medzikovic et al., [53] who examined the impact of hypertension on pulmonary HGF levels in rats. They observed increased *HGF* expression in hypertensive male rats, which was associated with improved clinical outcomes. However, this effect was absent in females, in whom no increase in HGF was detected and a worse clinical status was reported [53]. The absence of a positive correlation between HGF and hypertension in our study may be related to the fact that our study population consisted exclusively of women. However, this hypothesis should be verified in an independent cohort, including samples obtained from male subjects.

Increased expression of *FGFR1* and *FGFR2* was observed. However, activation of FGF signaling is generally not considered beneficial in the context of hypertension. This pathway has been implicated in the development of atherosclerosis. Furthermore, FGF signaling has been shown to reduce the contractility of primary aortic smooth muscle cells and promote a pro-inflammatory environment [54].

The hypertensive environment constitutes a highly adverse setting for cellular homeostasis. Under such conditions, the induction of compensatory mechanisms capable of counteracting hypertension-induced damage and facilitating tissue repair, including enhanced proliferation, angiogenesis, and suppression of apoptosis and fibrosis, would be expected. However, our results suggest that these compensatory responses are not elicited in UC-MSCs and MM-MSCs. Instead, the observed downregulation of numerous growth factors may indicate an impairment of these protective mechanisms in the hypertensive environment.

To gain a holistic understanding of the downstream consequences of this growth factor deficiency, our global microarray and Gene Ontology (GO) enrichment analyses revealed a highly destructive and profibrotic transcriptomic cascade in the fetal-placental niche. The documented decrease in key mitogens (*EGF*, *HGF*) and the *FGFR1* receptor by qPCR likely deprives UC-MSCs of essential survival and motogenic signals. At the transcriptomic level of the immune system, this deprivation manifests as a profound silencing of the basal transcriptional apparatus, particularly evident in the decreased transcription by RNA polymerase II (GO:0006357) and severe downregulation of pathways related to cell migration and locomotion (GO:0016477).

As a result, instead of maintaining a physiological vasoprotective state, these chronically stressed stem cells likely alter their functional commitment. Deprived of normal growth factor signaling, they activate the canonical Wnt signaling pathway (GO:0090263), suggesting a potential transition toward a pathological phenotype of tissue fibrosis. This transition is highlighted by the significant expression of structural genes regulating the collagen-containing extracellular matrix (GO:0062023) and extracellular matrix organization (GO:0030198). Furthermore, the concomitant loss of genes responsible for negative blood pressure regulation (GO:0045776) strongly suggests that maternal hypertension permanently disrupts the mechanisms of vascular protection and perinatal stem cell regeneration. However, these are only hypotheses based on transcriptomic results; these studies require further confirmation with proteomic studies and functional analyses of these cells.

What we believe is important is that this profound suppression of the *EGF* and *FGFR1* axis in hypertensive UC-MSCs strongly mirrors our parallel findings on the direct impact of fetal acid-base balance on stem cell homeostasis [55]. Our accumulating data suggest that extracellular pH serves as a fundamental functional “molecular switch” for stem cell niches during the perinatal period. Maternal gestational hypertension is clinically known to induce placental insufficiency, often leading to subclinical fetal hypoxia, hypercapnia, and subsequent respiratory or metabolic acidosis. Given that our parallel functional models identified low pH and hypercapnia as direct inhibitors of *EGF* and *FGFR1* transcription, we tentatively hypothesize that the transcriptome suppression observed in hypertensive UC-MSCs is mechanistically driven by these localized, acidosis-induced changes in the fetal microenvironment.

Although the observed transcriptomic shifts in pathways associated with cell migration and extracellular matrix organization provide insights into the potential functional plasticity of UC-MSCs, it should be noted that these findings are based on gene expression profiling. The results suggest a predisposition toward altered migratory and angiogenic behavior; however, functional validation is required to confirm these physiological consequences.

### 3.3. Effect of Maternal Hypothyroidism on Gene Expression in UC-MSCs and MM-MSCs

In our previous studies on the effects of maternal hypothyroidism on UC-MSCs, using stringent FDR filtering, we identified specific structural changes targeting extracellular matrix reorganization through activation of the *MMP1*, *MMP10*, and *GREM1* genes [10]. However, as postulated at the time, the small sample size limited the ability to fully map the overarching regulatory cascades. In this study, by extending the transcriptomic analysis with systemic profiling (GO pathways) and implementing qPCR validation on a much larger cohort, we uncover a deeper, regulatory level of this cellular adaptation. Our statistically significant increase in the expression of key trophic factors—*EGF* and *IGF1*—at the mRNA level in UC-MSCs directly corresponds to the massive activation of intracellular signal transduction pathways (GO:0035556) and the regulation of primary metabolic processes (GO:0080090), revealed in the global microarray profile. This suggests that the maternal thyroid hormone deficiency is associated with a robust trophic compensation program in UC-MSCs, in which EGF- and IGF-1-dependent signaling axes may play the role of overarching mediators protecting fetal cell plasticity and survival. This embryonic defense response is particularly compelling when compared to the current state of knowledge.

Numerous clinical studies indicate a drastic decrease in systemic IGF-1 concentrations in hypothyroidism [56,57,58]. Given that IGF-1 is a fundamental regulator of fetal growth and closely correlates with growth hormone (GH) levels [57,59], selective overexpression of this gene in UC-MSCs suggests the initiation of an autonomous fetal rescue mechanism aimed at protecting the developing offspring from maternal hormonal deficits. A similar relationship applies to the epidermal axis; the literature demonstrates that patients with untreated hypothyroidism are characterized by significantly reduced plasma EGF concentrations, which normalize only after the implementation of hormone replacement [60]. The increased expression of *EGF* in umbilical cord cells observed in our study confirms this compensatory mobilization, although the available clinical data do not allow us to clearly determine whether this effect results from the physiology of pregnancy itself or is a secondary response to the pharmacological treatment administered to the mothers.

A completely different regulatory dynamic was observed during the lactation period, where the mRNA profile in MM-MSCs and protein concentrations in whole milk were assessed in parallel. In the hypothyroid group, a clear downward trend was observed for *IGF1* expression in cells and HGF concentration in lactate; these fluctuations approached significance but did not reach the full statistical threshold. The only fully significant, destructive change was a drastic reduction in VEGF protein concentration in whole milk from women with hypothyroidism. This result indicates a significant impairment of the pro-angiogenic potential of the milk of these patients, which is fully consistent with the endocrine consensus: thyroid hormones are key stimulators of angiogenesis, and their deficiency directly inhibits vessel density by suppressing *VEGF* expression [61,62]. Apart from this selective angiogenic dysfunction, maternal hypothyroidism did not exert a global, destructive effect on the overall molecular and protein profile of lactate. This observation is highly consistent with the results published by Paśko et al., who assessed the concentrations of IGF-1, EGF, NGF, and TGF-beta in colostrum and also found no significant differences between samples from healthy women and patients with diagnosed hypothyroidism [63]. In summary, these preliminary reports suggest that controlled maternal hypothyroidism does not radically modify the biology of MM-MSCs with respect to the growth factors studied.

However, due to the lack of direct functional tests, the ultimate biological consequences of these subtle transcriptional changes remain unknown, and the potential impact of hypothyroidism on other paracrine axes not included in this panel requires further verification.

### 3.4. Relationship Between the History of Miscarriages and Stillbirths and IGF1 Expression in MSCs

Insulin-like growth factor 1 (IGF-1) is a multifunctional coordinator of perinatal homeostasis. It exerts profound antiapoptotic effects, drives cell proliferation and differentiation, regulates glucose metabolism, and is a fundamental driver of fetal growth [57,59,64]. Crucially, our study reveals a striking, long-lasting impact of obstetric trauma on the maternal lactation niche in subsequent successful pregnancies. To properly interpret these results, a clear distinction must be made between the cellular memory observed in isolated stem cells and the systemic secretory memory present in the whole milk matrix.

Regarding maternal miscarriage history, our qPCR analysis revealed selective upregulation of *IGF1* and *EGF* transcripts in the isolated MM-MSC fraction. In contrast, this upregulation in cells did not alter the macrocomposition of whole milk, as free protein levels measured by ELISA remained unchanged. This biological dissociation suggests that previous miscarriages are likely associated with alterations in the intrinsic transcriptional machinery of lactation stem cells, without altering global glandular production. On the other hand, a history of stillbirth manifested as a systemic secretory phenomenon characterized by a significant, nearly threefold increase in mature IGF-1 protein levels in whole milk, accompanied by a borderline upward trend in HGF concentrations.

From a functional perspective, IGF-1 and its cascades are widely recognized as essential protective shields for both the fetus and the newborn. Wang et al. demonstrated that IGF-1 directly enhances the survival and fitness of UC-MSCs [65], while Huang et al. observed its potent mitogenic effects on MSCs proliferation [66]. Furthermore, IGF-1-stimulated bone marrow-derived MSCs exhibit increased secretion of interleukin-10 (IL-10), a major anti-inflammatory cytokine [67]. Given that localized anti-inflammatory environments are essential for successful embryo implantation and pregnancy maintenance–and deletion of the IGF-1 receptor (Igf1r) leads to infertility in mice [68]—the tightly coordinated upregulation of the IGF-1 axis observed in our cohort underscores its evolutionary importance. Because cord blood IGF-1 levels closely correlate with neonatal birth weight and stimulate the structural development of the fetal brain, lungs, and microvasculature [64,68,69,70], its abundance is highly sensitive to changes in the microenvironment, including nutrient availability, stress induced by preeclampsia [70], and inflammatory factors [64].

Our findings indicate that traumatic obstetric events, such as miscarriages and stillbirths, likely leave a lasting, systemic imprint on the mother’s body, manifesting as a significant remodeling of lactation in subsequent successful pregnancies. The milk of these women naturally becomes richer in key mitogenic and survival factors (IGF-1, HGF). In light of the DOHaD hypothesis, this can be interpreted as an evolutionary compensatory mechanism—the maternal “paracrine shield” is strengthened to maximize support for the trophic development and immunomodulatory protection of the subsequent newborn. At this point, our results, although very interesting, require further in-depth analyses and confirmation on larger cohorts.

### 3.5. Relationships Between miR-155 Expression and Growth Factors in MSCs

MSCs express microRNA (miR) signatures that fine-tune their immunomodulatory and paracrine profiles [71]. Among them, miR-155 stands out as a key regulator of the response and remodeling frequency. A statistically significant positive correlation was found between miR-155 expression and *IGF1* transcripts in MM-MSCs, which is consistent with the anti-inflammatory and cytoprotective nature of the IGF-1 cascade [67].

This molecular interaction supports our hypothesis of a protective, compensatory response activated in mothers with a history of obstetrical distress. The positive correlation between *IGF1* and miR-155 provides a mechanistic link to pregnancy risk. Schjenken et al. established that miR-155 is crucial for maternal-fetal identification genetic deletion of miR-155 in mouse models has been associated with an aberrant, hostile immune response that leads to pregnancy loss [72]. Consequently, the simultaneous increase in miR-155 and *IGF1* levels in MM-MSCs from women with miscarriages suggests that these two elements work in tandem, enhancing the anti-inflammatory properties of the lactation niche, further contributing to the adaptive mechanisms of their effects on coordinating and protecting pregnancies.

Furthermore, our analysis revealed a clear correlation between miR-155 and *EGF* expression in MM-MSCs. This finding is indirect, consistent with Yang et al.’s identification of the epidermal growth factor receptor (EGFR) as a direct posttranscriptional target of miR-155. Their functional models, which provide increased miR-155 output, directly inhibit *EGFR* expression, while miR-155 inhibition causes a reciprocal increase in EGFR production [73]. We note that EGF acts as a primary high-affinity ligand for EGFR, a negative correlation that we observed in our study, which is related to the feedback loop in the mammary gland, where miR-155 regulates the importance and availability of the trophic axis.

In turn, there is a correlation between miR-155 and *HGF* expression in MM-MSCs. While the literature directly describing the biochemical interaction between miR-155 and *HGF* remains sparse, this positive co-expression likely stems from their common response to microenvironmental exposure. Both miR-155 and *HGF* are classically induced by proinflammatory cytokine currents, oxidative stress, and hypoxia [74,75,76]. In this context, their regulation does not mean a direct transcriptional action, but an appropriately synchronized activation of the cellular pathway aimed at balancing inflammatory solutions (driven by HGF) with the activation of epigenetic tuning (indirectly via miR-155).

The clinical relevance of this miR in vascular pathologies is strongly supported by the literature, as circulating miR-155 may serve as a potential non-invasive biomarker of hypertension and target organ damage in patients with essential hypertension [77]. Furthermore, miR-155 has emerged as a key regulator in the development of the disease [78]. Combining these established systemic roles with our new correlation patterns provides a compelling, unified epigenetic mechanism explaining how maternal gestational hypertension remodels the lactation niche. In our previous study, partially involving the same clinical cohort, we demonstrated that maternal hypertension induces a profound and statistically significant reduction in miR-155 expression in the MM-MSC fraction [35]. When superimposed on the correlation matrix discovered in the present study, this miR reduction provides an elegant explanation for the bidirectional behavior of growth factors under hypertensive stress. Because miR-155 exhibits a strong and positive correlation with *IGF1* (r = 0.72) and *HGF* (r = 0.64), its systemic inhibition in hypertensive pregnancies logically drives a synchronized downregulation of these survival and angiogenic factors, leading to the decrease in paracrine activity observed in the milk matrix. On the other hand, the strong negative correlation between miR-155 and *EGF* (r = −0.51) suggests that under physiological conditions, this miR acts as a constitutive posttranscriptional brake on the epidermal axis.

Consequently, the reduced miR-155 levels associated with maternal hypertension may correlate with a release of this epigenetic brake, potentially facilitating a compensatory derepression and subsequent trend toward increased *EGF* transcription in MM-MSCs. This complex feedback loop suggests that miR-155 may function as a central molecular regulator, potentially influencing the balance between growth factor depletion and the activation of a protective trophic shield within the lactation niche in response to maternal vascular stress. Although the observed correlation patterns between miR-155 and its target growth factors suggest a potential role in the epigenetic fine-tuning of the lactational niche, these associations require experimental validation to confirm the underlying regulatory mechanism.

This study provides, to the best of our knowledge, the first comprehensive evidence for tissue-specific transcriptional and secretory reprogramming in perinatal stem cell niches (UC-MSCs and MM-MSCs) influenced by maternal metabolic and vascular complications, as well as her obstetric history.

Our results provide strong molecular support for the DOHaD paradigm. We observed that the in vivo maternal microenvironment—defined by systemic hypertension, hypothyroidism, and traumatic obstetric experiences (miscarriages, stillbirths)—leaves a “transcriptional imprint” in the offspring’s stem cells and in the mother’s lactating niches, persisting after the initial stressor subsides.

The application of GLMs enabled a more nuanced interpretation of our transcriptomic data. After adjusting for potential confounding factors, including pre-pregnancy BMI, maternal diabetes, and mode of delivery, we demonstrated that the observed molecular signatures in both UC-MSCs and the lactational niche were not merely secondary to differences in neonatal maturity or maternal metabolic status. Instead, the persistent statistical significance and substantial effect sizes associated with maternal hypertension and a history of stillbirth provide robust molecular evidence supporting the programming of stem cell niches during intrauterine development and their long-term persistence after birth, consistent with the DOHaD paradigm.

### 3.6. Strengths, Limitations, and Future Prospects of the Study

#### 3.6.1. Strengths of the Study

The overarching value of this study is its globally innovative nature and unique biological approach to analyzing perinatal niches. To our knowledge, this is the first study to attempt to directly compare the molecular reactivity of MM-MSCs and UC-MSCs. This allowed for the definition of their tissue-specific functional arsenals. Introducing two distinct maternal pathologies into the research model—gestational hypertension (generating vascular stress) and hypothyroidism (generating metabolic stress)—allowed for a multifaceted assessment of MSCs plasticity.

These conditions, as demonstrated by numerous reports, have a fundamental impact on fetal intrauterine programming, the newborn’s condition, and subsequent child development. The paper guides the reader through the full discovery process—from global microarray screening, through targeted quantitative mRNA validation (qPCR), to partial assessment of protein secretion (ELISA). Beyond standard single-level profiling, a key strength of this study is the multi-layered data integration.

By comparing our current growth factor profiles with previously established microRNA-155 signatures from the same clinical cohort [35], we were able to move from a purely descriptive correlation matrix to a functional, causally linked regulatory mechanism. The discovery of strong bidirectional correlations between miR-155 and mitogens (IGF1, HGF, and EGF) provides a pioneering potential epigenetic explanation for the associated remodeling and behavioral dynamics of the lactation niche in the context of maternal vascular stress.

A major strength of our study is the application of multivariable GLMs, which enabled the identification of independent predictors of molecular programming within perinatal stem cell niches while accounting for potential confounding factors. We acknowledge that, in subgroups with limited sample sizes (e.g., mothers with hypothyroidism), adjustment for multiple covariates resulted in the loss of statistical significance for certain markers, such as IGF1. Rather than refuting these initial observations, we interpret this finding as a consequence of reduced statistical power inherent to analyses of small subgroups. Accordingly, these results are presented cautiously as hypothesis-generating observations rather than definitive evidence of causal relationships, reflecting our commitment to a rigorous and balanced interpretation of the data.

#### 3.6.2. Study Limitations

We are fully aware of the methodological and bioinformatic limitations of our work, which necessitate caution when formulating final application hypotheses. First, our microarray analysis is purely exploratory in nature due to the small initial sample size used for screening (control n = 2, hypertension n = 2, hypothyroidism n = 3). Given this very small number of cases, applying statistical correction methods for multiple testing, such as the false discovery rate (FDR), would result in no significantly differentially expressed genes being identified. Therefore, the obtained microarray results served solely as a preliminary baseline tool to identify candidate genes for targeted qPCR validation on a larger cohort. Secondly, while applying the strict Bonferroni correction (*p* < 0.01) to our qPCR and ELISA analyses ensures conservative and robust conclusions, we acknowledge that in subgroups with relatively small sample sizes, this stringent threshold increases the risk of Type II errors. Consequently, several biologically relevant trends observed in our study lost formal statistical significance, underscoring the need for further validation in larger multi-center cohorts More rigorous and advanced bioinformatic analyses (including rigorous FDR corrections) for these hypothyroid conditions have been published by our team in separate, earlier studies.

Second, although proper molecular validation was performed on a much larger cohort of patients, the number of patients in some pathological subgroups remains low (hypertension: n = 8 for MM-MSCs and n = 7 for UC-MSCs; hypothyroidism: n = 12 for MM-MSCs and n = 14 for UC-MSCs). This limitation stems directly from the natural, population-based incidence of these complications in pregnant women meeting the rigorous study inclusion criteria (no comorbidities).

Another significant limitation is the lack of assessment of the protein profile for UC-MSCs and the limitation of ELISA analysis in milk to only three key factors. Importantly, no statistically significant correlations between mRNA levels and the protein concentration were demonstrated in lactate. This is most likely due to the asymmetry of the biological matrices studied. Gene expression was determined directly in the isolated, pure cell fraction (MM-MSCs), whereas the protein concentration (ELISA) was measured in a whole milk matrix. Whole milk contains a pool of proteins synthesized and secreted primarily by mammary epithelial cells and immune cells, which masks and makes the overall lactate secretory profile independent of the transcriptional activity of the stem cells themselves.

Furthermore, the conclusions drawn are based primarily on the transcriptional level (mRNA). In this project, we did not conduct direct functional tests (such as proliferation, scratch assay migration, or directional differentiation assays) that would clearly confirm whether the observed changes in gene expression (e.g., *EGF* or *IGF* axes) permanently and physically alter the cell phenotype. These limitations should be taken into account when generating hypotheses.

Furthermore, the observational nature of this study precludes the establishment of definitive causal relationships. Although our transcriptomic and secretory data suggest substantial alterations in the functional potential of MSCs, including impaired migratory and angiogenic capacity, these functional consequences are inferred from gene expression and secretome profiles rather than demonstrated experimentally. Therefore, the proposed mechanistic interpretations should be regarded as preliminary, and further functional validation is required to determine whether maternal pathologies translate into biologically meaningful alterations in the regenerative capacity of these cells.

#### 3.6.3. Future Perspectives

The presented results, despite their limitations, constitute a valuable and unique starting point for further, advanced research into the mechanisms by which the maternal environment reprograms stem cells in vivo. The next logical research step will be to subject isolated cell lines (from both umbilical cords and the milk of patients with hypertension/thyroidism) to in vitro functional tests to assess their actual regenerative and migratory capacity. Plans are underway to expand the study to include assessment of the full secretome (secretory protein profile) of the cultured cells, which will overcome the problem of background noise present in whole milk. The most promising perspective is long-term clinical follow-up of children born from these pregnancies and correlation of the molecular profile of their perinatal stem cells (particularly the GDNF/NGF neurotrophic axis) with the subsequent neurodevelopmental and metabolic status of the newborn, which will allow for full verification of the DOHaD hypothesis.

## 4. Materials and Methods

### 4.1. Materials

The study was conducted using UC-MSCs and MM-MSCs stem cells. Umbilical cords were collected from 51 women, and breast milk from 78 women who gave birth at the Clinic of Obstetrics and Pregnancy Pathology of the Independent Public Clinical Hospital No. 1 in Lublin. The cords were collected immediately after delivery. Breast milk was obtained 2–3 days after delivery.

The study was conducted in accordance with the resolution of the Bioethics Committee of the Medical University of Lublin, No. KE_0254_7_01_2023. All study participants provided informed consent to the collection of biological material for research purposes.

Participants were also informed about the study’s aims and had the opportunity to contact the researchers with any questions or concerns. Analyses were performed for the entire cohort as well as in the following subgroups: (1) healthy controls, (2) patients with hypertension (blood pressure above 140/90 mmHg), and (3) patients with hypothyroidism diagnosed based on TSH levels [79].

### 4.2. Isolation of MSCs, Cell Culture Establishment and Flow Cytometry

MM-MSCs were isolated using the method described by Hassiotou et al. [80] The colostrum cell fraction (containing MM-MSCs) was isolated from 5 mL of whole human milk by centrifugation for 20 min at 805× *g* at 15 °C. The resulting cell pellet was separated from the supernatants and lipid phase and then washed twice with phosphate-buffered saline (PBS, Biomed, Poland) to eliminate protein contaminants [80,81,82,83]. An attempt was made to isolate the mesenchymal stem cell fraction (MM-MSCs) from all 78 samples, but samples from 54 patients were qualified for the final analysis due to the sufficient number of cells for analysis (n = 54).

The umbilical cord was first mechanically cut into small pieces using a sterile disposable scalpel. The obtained fragments were then subjected to enzymatic digestion with type I collagenase (Gibco by Life Technologies, Grand Island, NY, USA) for 3 h. Subsequently, the material was filtered, and the resulting suspension was centrifuged. The cell pellet obtained from both breast milk and the digested umbilical cord was used to establish cell cultures, which were maintained for 24 h.

Short-term culture was performed under plastic adhesion conditions in DMEM/F-12 medium (Corning, Manassas, VA, USA). The medium was supplemented with 10% FBS serum (ATCC, Teddington, UK) and 1% antibiotic mixture (penicillin-streptomycin) (Sigma-Aldrich, Jerusalem, Israel). The cells were incubated at 37 °C in an atmosphere containing 15% O_2_ and 5% CO_2_ [10,11,12,84,85,86,87].

To confirm the successful isolation of MSCs, the adherent properties of the obtained cells, their morphology, and their ability to form clusters were assessed using inverted light microscope, (Olympus CKX 41 and Olympus XC50 camera, Olympus, Tokyo, Japan) as well as their immunophenotype using a Navios flow cytometer (BeckmanCoulter, Indianapolis, IN, USA). The presence of surface antigens was assessed by flow cytometry: CD45, CD73, CD90, CD105, CD146, CD31, and CD14/CD19 (Supplementary Figure S1).

#### 4.2.1. Immunophenotypic Analysis by Flow Cytometry

Primary cultures of the isolated cells were subjected to immunophenotypic validation to confirm their mesenchymal origin. Immunophenotyping was performed using a flow cytometer (Navios, Beckman Coulter, Indianapolis, IN, USA). To specifically identify the MSCs population, the following fluorochrome-conjugated monoclonal antibodies were used: CD90-FITC, CD73-PE, CD34-ECD, CD146-PC5.5, CD105-PC7, CD31-Pacific Blue (PB), CD45-APC-A750, and CD14/CD19-Krome Orange (KRO). Data acquisition was performed using the Navios system software (v1.2), and subsequent data analysis was conducted using Kaluza Analysis Software (v2.1.1, Beckman Coulter).

#### 4.2.2. Gating Strategy

A sequential gating strategy was applied to ensure high sample purity and to exclude non-mesenchymal cell contaminants (Supplementary Figure S1). First, singlet events were identified by gating based on the forward scatter intensity (FS INT) versus either time or forward scatter peak (FS Peak), thereby excluding doublets and cellular aggregates.

Cell type-specific gating strategies were subsequently applied. For Wharton’s jelly-derived MSCs (UC-MSCs), cells were initially identified based on the co-expression of classical mesenchymal markers CD90 and CD73. Within this population, a negative gate was applied to exclude hematopoietic cells lacking CD34 expression.

For MM-MSCs, analysis was first restricted to singlets. Hematopoietic contamination was then excluded by gating on the CD45^−^ population. Within this non-hematopoietic fraction, expression of the perivascular mesenchymal marker CD146 was confirmed together with the absence of the endothelial marker CD31 (CD31^−^CD146^+^). Finally, the mesenchymal stromal cell phenotype was confirmed by identifying the CD90^+^CD105^+^CD73^+^ population and verifying the absence of additional lineage markers, including CD14/CD19 (CD14^−^/CD19^−^) and CD34.

Details regarding the procedures, equipment used, and reagents applied have been described in previous publications [10,11,12,81,82,83,84,85,86,87,88].

### 4.3. RNA Isolation and qPCR

After confirming the presence of MSCs, RNA isolation was initiated. A modified procedure proposed by Chomczyński and Sacchi was used. Total cellular RNA isolation from primary UC-MSCs cultures was performed using the TRIzol reagent (classical guanidinium-thiocyanato-phenol-chloroform extraction). After lysate extraction and the addition of chloroform, the aqueous phase was collected, from which RNA was precipitated with isopropanol, and the resulting pellet was purified in 75% ethanol. Details are described in a previous publication [11,12,84].

Isolation of nucleic acids from the cellular fraction of human milk (MM-MSCs) was performed using a column technique using the mirVana™ miRNA Isolation Kit (Thermo Fisher Scientific, Waltham, MA, USA) according to the manufacturer’s standard recommendations [35,83].

The obtained material was subjected to quantitative and qualitative analysis using a NanoDrop 2000c spectrophotometer (Thermo Fisher Scientific, Waltham, MA, USA).

Subsequently, RNA was reverse-transcribed into cDNA using a commercial High-Capacity cDNA Reverse Transcription Kit (Thermo Fisher Scientific, Waltham, MA, USA) containing an RNase inhibitor. Reverse transcription (RT) was performed on a 1 µg total RNA matrix. The thermal profile of the reaction included sequential incubation at 25 °C for 10 min, 37 °C for 120 min, and a final enzyme inactivation at 95 °C for 5 min.

Gene expression levels were analyzed on the StepOnePlus system using gene-specific molecular probes and the TaqMan Gene Expression Master Mix. PCR was performed using a 10-min initial denaturation at 95 °C, followed by 40 two-step cycles (15 s at 95 °C and 60 s at 60 °C). The cDNA was used to assess the expression of the following genes: *EGF*, *FGF2*, *FGFR1*, *FGFR3*, *GDNF*, *HGF*, *IGF1*, *NES*, *NGF*, and *PGF* by means of qPCR. Additionally, in human milk, miR-155 expression was evaluated.

The mRNA expression levels of human growth and neurotrophic factors were quantified by qPCR using pre-designed TaqMan Gene Expression Assays *EGF:* Hs0199990_m1 (NM_001178130), *FGF2:* Hs00266645_m1 (NM_002006), *FGFR1:* Hs00241111_m1 (NM_001174063), *FGFR3:* Hs00179829_m1 (NM_000142), *GDNF:* Hs00181185_m1 (NM_000514), *HGF:* Hs00300159_m1 (NM_000601), *IGF1:* Hs00153126_m1 (NM_001111283), *NES:* Hs04187831_g1 (NM_006617), *NGF:* Hs01113193_m1 (NM_002506), *PGF:* Hs00182176_m1 (NM_001207012), *GAPDH:* Hs99999905_m1 (NM_001289746) as an endogenous control (Thermo Fisher Scientific, Waltham, MA, USA). In the study material, we also assessed miR-155 expression in some MM-MSCs according to the publication [35]. The detailed physiological functions and characteristics of each evaluated gene are systematically summarized in Table 14.

Gene expression (RQ) was calculated based on the formula proposed by Livak et al.: RQ = 2^−ddCt^ [89]. Glyceraldehyde-3-phosphate dehydrogenase (*GAPDH*) was used as a stable internal reference gene (endogenous control). Relative transcript expression levels were calculated based on cycle cutoff values (CT) in Expression Suite Software (LifeTechnology). Information regarding the equipment and reagents used is provided in the publication [10,11,12,81,82,83,84,85,86,87,88].

### 4.4. Screening on Expression Microarrays (UC-MSC Cells)

Due to the exploratory nature of this screening step, microarray profiling was performed on a selected sub-cohort of samples: physiological pregnancy/control (n = 2), gestational hypertension (n = 2), and maternal hypothyroidism (n = 3).

Global assessment of gene expression profile in UC-MSCs was performed using the Affymetrix GeneChip Human Gene 2.0 ST Array oligonucleotide microarray system. Intact RNA samples with a RIN greater than 7.0 were accepted for analysis. Microarray scanning was performed using the Affymetrix GeneChip Scanner 3000 7G (Affymetrix, Santa Clara, CA, USA) equipped with Workstation and AutoLoader modules. Raw data from CEL files were preprocessed in the Affymetrix GeneChip Command Console Software (v4.0, AGCC). Mapping and differential gene identification (DEGs) between groups were performed in the Affymetrix Transcriptome Analysis Console (TAC). Significance criteria for regulated genes were an absolute log2 fold change > 1 and a *p*-value < 0.05 [10,55].

### 4.5. Evaluation of the Protein Concentration in Whole Milk by ELISA Method

The assessment of the growth factor concentration at the protein level in the whole milk matrix was performed for the full study cohort (n = 78). The concentrations of growth factors IGF-1, VEGF, and HGF were determined in whole milk using an ELISA assay. Initially, milk samples were twice diluted. Subsequently, using the Human IGF-1 Rapid ELISA Kit EELR024 (Invitrogen, Waltham, MA, USA), Human VEGF ELISA Kit KHG0111 (Invitrogen), and Human HGF Instant ELISA™ Kit BMS2069INST (Invitrogen), the concentrations of individual proteins were measured according to the manufacturer’s protocol. The analysis was performed using an ETI MAX 3000 analyzer (DiaSorin, Madison, WI, USA).

### 4.6. Statistical and Bioinformatics Analysis

Statistical analysis and visualization of the obtained results were performed using dedicated bioinformatics and statistical software, with a statistical significance level of *p* = 0.05.

#### 4.6.1. Expression Microarray Analysis

Initial identification of differentially expressed genes (DEGs) in umbilical cord tissue (UC-MSCs) was performed using the Transcriptome Analysis Console (TAC) Software (Affymetrix/Thermo Fisher Scientific). The cutoff criteria for exploratory analysis were a raw (uncorrected) *p* value of <0.05 and a fold change of expression (FC) > 2. Due to the very small sample size in this initial screening phase, statistical methods for correcting for multiple testing (such as false discovery rate (FDR)) were not used, as this would predictably result in failure to identify significantly differentially expressed genes. Therefore, the microarray data were used solely as an exploratory tool to select promising candidate genes for further rigorous quantitative validation (qPCR) in a larger cohort. Pathway enrichment analysis for functional and biological ontologies (GO, KEGG, WikiPathways) was performed using g:Profiler [90], in which statistical significance was determined by an adjusted *p*_adj value of <0.05 using the default g:SCS method.

#### 4.6.2. qPCR Data Analysis and Hierarchical Clustering

Volcano plot visualization of gene expression profiles and relative transcript quantification were performed using ExpressionSuite Software (Life Technologies). Hierarchical clustering analysis of phenotypic samples (MM-MSCs vs. UC-MSCs) and the studied genes was presented in heatmaps generated based on global Delta CT values using Euclidean distance as a similarity measure and Complete Linkage as a cluster agglomeration algorithm.

#### 4.6.3. Statistical Verification (qPCR, ELISA, and Clinical Correlations)

Actual statistical verification of quantitative data from qPCR and ELISA tests was performed in Statistica (v13.5, StatSoft, Inc., Tulsa, OK, USA). In the first step, the normality of the distribution of variables was assessed using the Shapiro–Wilk test.

Relative gene expression values were logarithmically transformed (log_10RQ). If a normal distribution was found, parametric significance tests (Student’s *t*-test for independent samples) were used to assess differences between the study and control groups. Genes that did not exhibit a normal distribution or showed no expression in selected replicates were analyzed using nonparametric tests. For ELISA data, due to the non-normal distribution of raw protein concentrations (VEGF, HGF, IGF-1), a comparative analysis of factor concentrations in human milk between clinical groups was performed using the nonparametric Mann–Whitney test.

The correlation between milk protein concentrations (ELISA), umbilical cord gene expression levels (qPCR), and selected clinical parameters and the patients’ obstetric history (including the number of previous miscarriages and stillbirths) was assessed using the nonparametric Spearman rank correlation coefficient (r).

To minimize the risk of false positive results (Type I error) resulting from multiple comparisons during the quantitative validation phase, a Bonferroni correction was applied to the qPCR and ELISA data. Given that target panels of predefined genes/proteins were analyzed, the conventional significance level of alpha = 0.05 was adjusted accordingly. Therefore, statistical significance for these analyses was defined by a stringent threshold of *p* < 0.01. Multivariate analyses were performed using general linear models (GLM) adjusted for clinical variables. A two-sided *p* value of <0.05 was maintained in the GLM analyses.

To rigorously evaluate the independent effects of maternal pathological conditions and obstetric history on the transcriptomic and secretory profiles of stem cells, multivariable analyses were performed using GLMs. For UC-MSCs, the models were adjusted for gestational age, neonatal birth weight, mode of delivery, and maternal diabetes. For the human milk niche (MM-MSCs transcriptomic analyses and ELISA measurements of whole milk), the GLMs were adjusted for pre-pregnancy BMI, maternal diabetes, and mode of delivery to account for major metabolic and obstetric confounders while avoiding model overfitting. A two-sided *p* value < 0.05 was considered statistically significant, and effect sizes were quantified using partial eta squared (ηp^2^).

## 5. Conclusions

This study represents a pioneering, multi-layered comparison of the molecular reactivity of UC-MSCs and MM-MSCs under pathological maternal conditions. Our findings lead to the following few conclusions.

Perinatal stem cell niches possess distinct, built-in functional arsenals at the transcript level. UC-MSCs demonstrate distinct structural and vascular commitments tailored to fetoplacental support, whereas MM-MSCs exhibit an epithelial-trophic protective signature potentially adapted for neonatal gastrointestinal homeostasis.

Maternal complications are associated with alterations in these transcriptome and secretory profiles in a highly asymmetric, tissue-specific manner. Gestational hypertension correlates with a transcriptomic shift in the UC-MSCs niche toward an exhausted, fibrotic signature, while simultaneously being associated with a trend toward a compensatory trophic response in milk cells. In turn, maternal hypothyroidism is linked to a strong transcriptomic rescue response in UC-MSCs, as well as a potential paracrine translational deficit within the lactational matrix of MM-MSCs.

A traumatic maternal obstetric history (miscarriages and stillbirths) may leave a lasting cellular and secretory imprint on subsequent lactations, marked by a trend toward the coordinated mobilization of vital mitogens (IGF-1, HGF). Furthermore, the expression of miR-155 correlates with these growth factor profiles, suggesting a potential role in epigenetic regulation.

Furthermore, using multivariable modeling, we demonstrated that maternal vascular stress (hypertension) and obstetric adversity (a history of stillbirth) act as independent statistical predictors of perinatal stem cell plasticity. These findings underscore the clinical relevance of considering these maternal conditions when evaluating the suitability of perinatal stem cell sources for biobanking and regenerative medicine applications.

In summary, this work suggests that the maternal intrauterine, metabolic, and hormonal environment is persistently associated with the molecular phenotype of perinatal stem cell niches. This remarkable cellular plasticity provides molecular support for the DOHaD paradigm, indicating that the functional pattern of offspring stem cells may directly reflect the maternal somatic health status.

## Figures and Tables

**Table 1:** Demographic characteristics, anthropometric parameters, and clinical profiles of mothers and newborns in the study groups.

Parameter	MM-MSCs (n = 78 ELISA/n = 54 qPCR) *			UC-MSCs (n = 51)		
	Control (n = 50 ELISA/n = 34 qPCR)	Hypertension (n = 8 ELISA and qPCR)	Hypothyroidism (n = 20 ELISA/n = 12 qPCR)	Control (n = 30)	Hypertension (n = 7)	Hypothyroidism (n = 14)
Mothers’ age [years]	30.51 ± 5.51/ 31.33 ± 6.05	30.38 ± 1.85	29.9 ± 4.10/ 29.44 ± 3.94	32.61 ± 4.34	34 ± 6.69	32.36 ± 5.72
Number of pregnancies	2.11 ± 1.27/ 2.1 ± 1.16	2 ± 1.15	1.89 ± 0.94/ 1.75 ± 0.701	2.22 ± 1.17	2.83 ± 1.72	2 ± 1.109
GA [weeks]	39.29 ± 1.23/ 39.13 ± 1.33	37.25 ± 2.76	37.8 ± 2.02/ 37.67 ± 2.74	38.44 ± 1.65	37.66 ± 2.07	38.28 ± 1.46
Newborn weight [g]	3516 ± 509/ 3448 ± 504	2946 ± 857.95	3193 ± 578/ 3012 ± 778	3336 ± 380	3040 ± 356	3118 ± 315
Umbilical cord blood pH	7.31 ± 0.06/ 7.32 ± 0.07	7.297 ± 0.071	7.32 ± 0.05/ 7.32 ± 0.048	7.322 ± 0.09	7.317 ± 0.027	7.36 ± 0.056

Data are presented as mean ± standard deviation (SD). Patients from whom stem cells (UC-MSCs and MM-MSCs) were isolated were assigned to three independent clinical subgroups: a control group (normal pregnancies), a group with gestational hypertension, and a group with hypothyroidism. Abbreviations: GA—gestational age; MM-MSC—maternal milk-derived mesenchymal stem cells; UC-MSC—umbilical cord-derived mesenchymal stem cells. * Of the 78 milk samples, a sufficient fraction of MM-MSCs for high-quality RNA isolation was obtained from 54 patients.

**Table 2:** Gene Ontology (GO) functional classification of genes showing upregulation in UC-MSCs under the influence of maternal gestational hypertension.

Source	Term ID	Term Name	*p*adj
GO:CC	GO:0062023	collagen-containing extracellular matrix	1.204 × 10^−9^
GO:BP	GO:0030334	regulation of cell migration	1.410 × 10^−7^
GO:MF	GO:0005539	glycosaminoglycan binding	1.086 × 10^−5^
GO:CC	GO:0043202	lysosomal lumen	1.159 × 10^−5^
GO:MF	GO:0005201	extracellular matrix structural constituent	4.353 × 10^−5^
GO:BP	GO:0023057	negative regulation of signaling	8.115 × 10^−5^
GO:CC	GO:0005796	Golgi lumen	1.984 × 10^−4^
GO:BP	GO:0035295	tube development	2.202 × 10^−4^
GO:CC	GO:0030054	cell junction	2.203 × 10^−4^
GO:CC	GO:0005576	extracellular region	3.365 × 10^−4^
GO:BP	GO:0030198	extracellular matrix organization	8.273 × 10^−4^
GO:MF	GO:0098772	molecular function regulator activity	2.273 × 10^−3^
GO:CC	GO:0009986	cell surface	5.564 × 10^−3^
GO:MF	GO:0005515	protein binding	6.821 × 10^−3^
GO:BP	GO:0007155	cell adhesion	9.106 × 10^−3^
GO:BP	GO:0065007	biological regulation	1.254 × 10^−2^
GO:BP	GO:0090263	positive regulation of canonical Wnt signaling pathway	1.308 × 10^−2^
GO:MF	GO:0043878	glyceraldehyde-3-phosphate dehydrogenase (NAD^+^) activity	1.609 × 10^−2^
GO:BP	GO:0033012	muscle system process	2.925 × 10^−2^
GO:BP	GO:0032502	developmental process	3.316 × 10^−2^

**Table 3:** Gene Ontology (GO) functional classification for genes exhibiting downregulated expression in UC-MSCs under the influence of maternal gestational hypertension.

Source	Term ID	Term Name	*p*adj
GO:MF	GO:0005515	protein binding	3.564 × 10^−27^
GO:BP	GO:0065007	biological regulation	3.327 × 10^−23^
GO:CC	GO:0016020	membrane	2.002 × 10^−18^
GO:CC	GO:0005737	cytoplasm	2.547 × 10^−9^
GO:MF	GO:0003700	DNA-binding transcription factor activity	1.063 × 10^−7^
GO:MF	GO:0000977	RNA polymerase II transcription regulatory region sequence-specific DNA binding	3.429 × 10^−5^
GO:CC	GO:0042995	cell projection	4.218 × 10^−4^
GO:BP	GO:0006357	regulation of transcription by RNA polymerase II	6.493 × 10^−4^
GO:MF	GO:0022857	transmembrane transporter activity	8.907 × 10^−4^
GO:BP	GO:0044281	small molecule metabolic process	1.434 × 10^−3^
GO:CC	GO:0005576	extracellular region	1.735 × 10^−3^
GO:BP	GO:0055085	transmembrane transport	1.873 × 10^−3^
GO:CC	GO:0030054	cell junction	1.893 × 10^−3^
GO:BP	GO:0016477	cell migration	4.551 × 10^−3^
GO:BP	GO:0050851	antigen receptor-mediated signaling pathway	7.692 × 10^−3^
GO:BP	GO:0045776	negative regulation of blood pressure	8.927 × 10^−3^
GO:CC	GO:0031982	vesicle	1.244 × 10^−2^
GO:CC	GO:0009986	cell surface	1.325 × 10^−2^
GO:CC	GO:0150034	distal axon	2.120 × 10^−2^
GO:CC	GO:0000785	chromatin	2.320 × 10^−2^
GO:MF	GO:0038023	signaling receptor activity	3.066 × 10^−2^
GO:MF	GO:0036094	small molecule binding	3.242 × 10^−2^
GO:BP	GO:0040011	locomotion	4.928 × 10^−2^

**Table 4:** Changes in the relative expression of the studied genes (qPCR) in UC-MSC cells, comparing the group with maternal gestational hypertension with the control group.

UC-MSCs					
Gene	Hypertension n = 7		Control n = 30		*p*-Value
	Mean	SD	Mean	SD	
logRQ *EGF* ↓	−0.470	0.382	−0.098	0.353	0.041
logRQ *FGFR1* ↓	−0.422	0.334	−0.061	0.221	0.006 *
logRQ *HGF* ↓	−0.560	0.533	−0.006	0.525	0.02

Data are presented as the mean logarithmic transformation of relative expression (log_10RQ ± SD). Statistical significance between the gestational hypertension group and the control group (physiological pregnancies) was assessed using the parametric Student’s *t*-test for independent samples. * *p* values < 0.01 were considered statistically significant following Bonferroni adjustment. ↓ indicates a statistically significant decrease or a downward trend in the study group compared to the control group.

**Table 5:** Results of pathway enrichment analysis for downregulated genes in UC-MSCs derived from patients with hypothyroidism compared with healthy controls.

Source	Pathway/Process Name	Term ID	Adjusted *p*-Value (*p*adj)	Downregulated Genes
KEGG	ABC transporters	KEGG:02010	3.359 × 10^−2^	*ABCA6*, *ABCA8*, *ABCA9*
CORUM	G protein complex (BTK, GNG1, GNG2)	CORUM:1615	1.771 × 10^−4^	*BTK*

**Table 6:** Top 10 statistically most significant enriched categories among upregulated genes in UC-MSCs derived from patients with hypothyroidism compared with healthy controls.

Source	Pathway/Process Name	Term ID	Adjusted *p*-Value (*p*adj)	Biological Significance
GO:CC	Cytoplasm	GO:0005737	6.78 × 10^−56^	Indicates that many differentially expressed proteins are localized in the cytoplasm.
GO:MF	Protein binding	GO:0005515	7.90 × 10^−53^	Reflects extensive protein interaction networks and signaling activity.
GO:CC	Cytosol	GO:0005829	9.48 × 10^−32^	Suggests enrichment of proteins functioning in the soluble intracellular compartment.
GO:CC	Nucleoplasm	GO:0005654	3.44 × 10^−26^	Indicates altered expression of genes involved in nuclear functions and transcriptional regulation.
GO:BP	Biological regulation	GO:0065007	1.28 × 10^−20^	Demonstrates broad alterations in cellular regulatory mechanisms.
GO:BP	Regulation of biological process	GO:0050789	1.53 × 10^−19^	Suggests widespread modulation of biological activities.
GO:BP	Regulation of primary metabolic process	GO:0080090	2.06 × 10^−19^	Indicates changes in metabolic regulation potentially linked to thyroid hormone deficiency.
GO:BP	Regulation of response to stimulus	GO:0048583	8.68 × 10^−19^	Suggests altered cellular responsiveness to environmental and physiological signals.
GO:BP	Macromolecule modification	GO:0043412	2.68 × 10^−18^	Reflects changes in protein processing and post-translational modifications.
GO:BP	Positive regulation of biological process	GO:0048518	6.44 × 10^−18^	Indicates activation of pathways promoting cellular activity and adaptation.

**Table 7:** Changes in the relative expression of the studied genes (qPCR) in UC-MSCs: comparison of the group with maternal hypothyroidism with the control group.

UC-MSCs					
Gene	Hypothyroidism n = 14		Control n = 30		*p*-Value
	Mean	SD	Mean	SD	
logRQ *IGF1* ↑	−0.406	0.762	−0.824	0.696	0.028
logRQ *EGF* ↑	0.126	0.427	−0.201	0.405	0.046

↑ indicates a statistically significant increase or an upward trend.

**Table 8:** General linear model (GLM) analysis assessing the independent effects of maternal conditions and clinical covariates on gene expression (logRQ) in UC-MSCs.

UC-MSCs					
Dependent Variable	Main Factor	Main Factor Effect F (df1, df2)	*p*-Value	Effect Size (ηp^2^)	Covariates *p*-Values (Birth Weight/GA/Mode/Diabetes)
Maternal Hypertension Models					
logRQ *EGF*	Hypertension	F(1, 41) = 4.75	0.035 *	0.104	0.374/0.926/0.021/0.534
logRQ *FGFR1*	Hypertension	F(1, 43) = 11.54	0.001 *	0.212	0.155/0.559/0.353/0.941
logRQ *HGF*	Hypertension	F(1, 42) = 6.85	0.012 *	0.140	0.207/0.705/0.970/0.803
Maternal Hypothyroidism Models					
logRQ *EGF*	Hypothyroidism	F(2, 40) = 3.52	0.039 *	0.150	0.230/0.731/0.061/0.063
logRQ *IGF1*	Hypothyroidism	F(1, 42) = 1.87	0.179	0.043	0.685/0.874/0.406/0.423

* *p*-values < 0.05 were considered statistically significant for the multivariable general linear model (GLM) analysis.

**Table 9:** Changes in the relative expression of the studied genes (qPCR) and the studied proteins (ELISA) in MM-MSCs: comparison of the group with maternal hypertension with the control group.

MM-MSCs					
Gene	Hypertension n = 8		Control n = 34		*p*-Value
	Mean	SD	Mean	SD	
logRQ *EGF* ↑	0.816	0.265	0.109	0.177	0.039
logRQ *FGFR3* ↑	−0.153	0.877	−0.864	0.745	0.03
logRQ *HGF* ↓	−1.021	0.316	−0.009	0.622	0.024
logRQ *IGF1* ↓	−1.431	0.620	−0.592	0.526	0.042
logRQ *NES* ↑	−0.230	0.656	−1.229	0.823	0.003 *
**Protein**	**Hypertension n = 8**		**Control n = 50**		***p*-Value**
	**Mean**	**SD**	**Mean**	**SD**	
VEGF [pg/mL] ↓	467.173	330.661	732.210	454.490	0.262
HGF [pg/mL] ↓	139.900	168.617	905.110	1806.952	0.294
IGF [ng/mL] ↓	15.701	24.024	26.090	52.830	0.556

* *p* values < 0.01 were considered statistically significant following Bonferroni adjustment. ↑ indicates a statistically significant increase or an upward trend. ↓ indicates a statistically significant decrease or a downward trend in the study group compared to the control group.

**Table 10:** Changes in the relative expression of the tested genes (qPCR) and the tested proteins (ELISA) in MM-MSCs: comparison of the group with maternal hypothyroidism with the control group.

MM-MSCs					
Gene	Hypothyroidism n = 12		Control n = 34		*p*-Value
	Mean	SD	Mean	SD	
logRQ *EGF* ↑	0.578	0.381	0.109	0.177	0.049
logRQ *HGF* ↓	−0.442	0.798	−0.009	0.622	0.046
logRQ *IGF1* ↓	−1.307	1.086	−0.592	0.526	0.042
**Protein**	**Hypothyroidism n = 20**		**Control n = 50**		***p*-Value**
	**Mean**	**SD**	**Mean**	**SD**	
VEGF [pg/mL] ↓	452.84	237.05	732.210	454.490	0.029
HGF [pg/mL] ↓	305.49	435.67	905.110	1806.952	0.080
IGF [ng/mL] ↓	3.89	3.67	26.090	52.830	0.06

↑ indicates a statistically significant increase or an upward trend. ↓ indicates a statistically significant decrease or a downward trend in the study group compared to the control group.

**Table 11:** Changes in the relative expression of the tested genes (qPCR) and tested proteins (ELISA) in MM-MSCs: comparison of the group with a history of stillbirths and miscarriages in the obstetric history compared to the group without a history of miscarriages and stillbirths.

MM-MSCs Obstetric History					
History of Miscarriages					
Gene	Yes n = 12		No n = 42		*p*-Value
	Mean	SD	Mean	SD	
logRQ *EGF* ↑	0.569	1.408	0.109	0.177	0.026
logRQ *FGFR1* ↑	0.602	1.094	0.246	0.334	0.066
logRQ *IGF1* ↑	0.399	0.145	0.145	0.145	0.022
**Protein**	**Yes n = 22**		**No n = 56**		***p*-Value**
	**Mean**	**SD**	**Mean**	**SD**	
IGF [ng/mL]	27.221	54.562	23.597	35.792	0.724
HGF [pg/mL]	770.937	1682.588	737.093	1563.913	0.932
VEGF [ng/mL]	576.354	331.254	657.493	441.097	0.433
History of Stillbirths					
**Protein**	**Yes n = 6**		**No n = 72**		***p*-Value**
	**Mean**	**SD**	**Mean**	**SD**	
IGF [ng/mL] ↑	60.124	105.488	22.311	33.924	0.045
HGF [pg/mL] ↑	2059.342	3006.737	663.759	1448.638	0.056
VEGF [pg/mL] ↑	909.923	740.602	619.403	387.848	0.130

↑ indicates a statistically significant increase or an upward trend.

**Table 12:** GLM analysis assessing the independent effects of maternal conditions and obstetric history on the transcriptomic (logRQ) and secretory (ELISA) profiles of the lactational niche.

MM-MSCs					
Dependent Variable	Main Factor	Main Factor Effect F (df1, df2)	*p*-Value	Effect Size (ηp^2^)	Covariates *p*-Values (BMI/Diabetes/Mode of Delivery)
Maternal Hypertension Models					
logRQ *NES*	Hypertension	F(1, 47) = 8.22	0.006 *	0.149	0.817/0.522/0.264
logRQ *FGFR3*	Hypertension	F(1, 31) = 5.13	0.031 *	0.142	0.644/0.395/0.282
logRQ *HGF*	Hypertension	F(1, 26) = 4.60	0.041 *	0.150	0.441/0.730/0.482
logRQ *EGF*	Hypertension	F(1, 47) = 4.13	0.048 *	0.081	0.825/0.292/0.877
Whole Milk Secretion (Stillbirth History Model)					
HGF Protein	Stillbirths	F(1, 47) = 11.33	0.002 *	0.194	0.611/0.701/0.486
IGF Protein	Stillbirths	F(1, 45) = 10.35	0.002 *	0.187	0.186/0.394/0.919
VEGF Protein	Stillbirths	F(1, 47) = 4.84	0.033 *	0.093	0.380/0.246/0.072

* *p*-values < 0.05 were considered statistically significant for the multivariable general linear model (GLM) analysis.

**Table 13:** Integration of molecular data—a combination of global microarray screening (TAC/g:Profiler) with targeted quantitative validation (qPCR/ELISA) in UC-MSCs and MM-MSCs under the influence of maternal pathologies.

Factor/Gene	Effect in UC-MSCs (Microarray Analysis: TAC + g:Profiler)	Effect in UC-MSCs (qPCR)	Effect in MM-MSCs (qPCR/ELISA)
Hypertension			
*EGF*	↓ Downregulation of gene expression • Supporting pathways (DOWN): Regulation of transcription (GO:0006357), transcription factor activity (GO:0003700), cell migration (GO:0016477).	↓ Decrease (qPCR)	↑ Increase (qPCR)
*IGF1/IGF*	No significant changes (grey zone) • Supporting pathways (DOWN): General silencing of biological regulation (GO:0065007) and signaling receptor activity (GO:0038023).	No significant changes	↑Increase (qPCR)
*HGF*	↓ Decrease in expression of angiogenic receptors (*FGFR1*) and mitogens • Supporting pathways (DOWN): Cell migration (GO:0016477), locomotion (GO:0040011), negative regulation of blood pressure (GO:0045776).	↓ Decrease (qPCR)	↓ Decrease (qPCR)
*FGFR1*	↓ Downregulation of gene expression • Pathways (DOWN): Protein tyrosine kinase activity (GO:0004713), growth factor signaling cascades.	↓ Decrease (qPCR)	No significant changes
*FGFR3*	No significant changes	No significant changes	↑ Increase (qPCR)
*NES*	No significant changes • Supporting pathways (UP): Extracellular matrix organization (GO:0030198), collagen-containing ECM (GO:0062023)—fibrotic phenotype.	No significant changes	↑ Increase (qPCR)
Hypothyroidism			
*EGF*	Global increase in transcriptional background • Supporting pathways (UP): Intracellular signal transduction (GO:0035556), cellular response to stimulus (GO:0051716).	↑ Increase (qPCR)	↑ Increase (qPCR)
IGF1/IGF	↑ Strong upregulation of the entire axis (IGFL1, IGFL2, IGF1R) • Supporting pathways (UP): Regulation of primary metabolic processes (GO:0080090), RNA biosynthetic process (GO:2001141), nucleoplasm localization (GO:0005654).	↑ Increase (qPCR)	↓ Decrease (mRNA and protein)
VEGF, HGF	No changes for the genes themselves (massive derepression of the remaining background) • Supporting pathways (DOWN): Suppression of ATP-binding cassette transporters (ABCA6, ABCA8, ABCA9—KEGG:02010), lipid metabolic disorders.	No significant changes	↓ Decrease in protein (ELISA)

↑ indicates a statistically significant increase or an upward trend. ↓ indicates a statistically significant decrease or a downward trend in the study group compared to the control group.

**Table 14:** Functions of selected growth factors secreted by mesenchymal stem cells.

Gene	Full Name	Functions	References
*EGF*	epidermal growth factor	proliferation, migration, soluble EGF does not induce differentiation but promotes the secretion of VEGF and HGF, tethered EGF promotes osteogenic differentiation, trophoblast development, regulation of homeostasis, embryonic development	[20,21,22,23]
*FGF2*	fibroblast growth factor 2	proliferation, self-renewal, angiogenesis, embryonic development, osteoblast differentiation, bone formation	[16,24]
*GDNF*	glial cell derived neurotrophic factor	differentiation, migration, neuronal regeneration, immunomodulation, increased dopamine uptake, neurite elongation and branching, neuroprotection	[17,29,30]
*HGF*	hepatocyte growth factor	migration, endothelial cell growth, antiapoptotic effects, vasodilation, inhibition of fibrosis, morphogenesis	[25,26]
*IGF1*	insulin like growth factor 1	proliferation, self-renewal, differentiation, migration, anti-apoptotic, postnatal growth, embryonic development, neuroprotection, angiogenesis	[16,17,18,19]
*NES*	nestin	proliferation, migration, self-renewal, marker of newly formed blood vessels, antioxidant effects, nervous system development, differentiation, fibrosis	[32,33,34]
*NGF*	nerve growth factor	stimulation of cell growth, differentiation, neuronal maturation, neuroprotection (particularly of cholinergic neurons), neuronal regeneration, immunomodulation	[17,29,30,31]
*PGF*	placental growth factor	angiogenesis, placenta development	[19,27,28]

## Data Availability

The original contributions presented in this study are included in the article/Supplementary Material. Further inquiries can be directed to the corresponding author.

## Supplementary Materials

The following supporting information can be downloaded at: https://www.mdpi.com/article/10.3390/ijms27188269/s1.

## References

[1] Liu J., Gao J., Liang Z., Gao C., Niu Q., Wu F., Zhang L. (2022). Mesenchymal stem cells and their microenvironment. Stem Cell Res. Ther..

[2] Hussein M. (2025). Advancing regenerative therapies with umbilical cord–derived mesenchymal stem cells: A review. Biomol. Biomed..

[3] Carp D.M., Liang Y. (2022). Universal or Personalized Mesenchymal Stem Cell Therapies: Impact of Age, Sex, and Biological Source. Cells.

[4] Zhidu S., Ying T., Rui J., Chao Z. (2024). Translational potential of mesenchymal stem cells in regenerative therapies for human diseases: Challenges and opportunities. Stem Cell Res. Ther..

[5] Costela-Ruiz V.J., Melguizo-Rodríguez L., Bellotti C., Illescas-Montes R., Stanco D., Arciola C.R., Lucarelli E. (2022). Different Sources of Mesenchymal Stem Cells for Tissue Regeneration: A Guide to Identifying the Most Favorable One in Orthopedics and Dentistry Applications. Int. J. Mol. Sci..

[6] Swain H.N., Boyce P.D., Bromet B.A., Barozinksy K., Hance L., Shields D., Olbricht G.R., Semon J.A. (2024). Mesenchymal stem cells in autoimmune disease: A systematic review and meta-analysis of pre-clinical studies. Biochimie.

[7] Chen H., Liu O., Chen S., Zhou Y. (2022). Aging and Mesenchymal Stem Cells: Therapeutic Opportunities and Challenges in the Older Group. Gerontology.

[8] Maged G., Abdelsamed M.A., Wang H., Lotfy A. (2024). The potency of mesenchymal stem/stromal cells: Does donor sex matter?. Stem Cell Res. Ther..

[9] Pestel J., Blangero F., Eljaafari A. (2023). Pathogenic Role of Adipose Tissue-Derived Mesenchymal Stem Cells in Obesity and Obesity-Related Inflammatory Diseases. Cells.

[10] Przywara D., Babiuch W., Petniak A., Piszcz B., Krzyżanowski A., Kondracka A., Kocki J., Gil-Kulik P. (2026). The Impact of Maternal Diabetes and Hypothyroidism on Signaling Pathway Activation and Gene Expression in Fetal Mesenchymal Stem Cells. Biomedicines.

[11] Bieńko K., Leszcz M., Więckowska M., Białek J., Petniak A., Szymanowski R., Wilińska A., Piszcz B., Krzyżanowski A., Kwaśniewska A. (2023). VEGF Expression in Umbilical Cord MSC Depends on the Patient’s Health, the Week of Pregnancy in Which the Delivery Took Place, and the Body Weight of the Newborn–Preliminary Report. Stem Cells Cloning Adv. Appl..

[12] Ozygała A., Wojciechowska G., Traczyk K., Przywara D., Petniak A., Szymanowski R., Wilińska A., Krzyżanowski A., Kwaśniewska A., Płachno B.J. (2023). Influence of Umbilical Cord Blood Biochemical Parameters and Disease Condition on the Expression of the TSG-6 Gene in Umbilical Mesenchymal Stem Cells. Med. Sci. Monit..

[13] Salaudeen M.A., Allan S., Pinteaux E. (2023). Hypoxia and interleukin-1-primed mesenchymal stem/stromal cells as novel therapy for stroke. Hum. Cell.

[14] Li H., Ji X.Q., Zhang S.M., Bi R.H. (2023). Hypoxia and inflammatory factor preconditioning enhances the immunosuppressive properties of human umbilical cord mesenchymal stem cells. World J. Stem Cells.

[15] Faa G., Pichiri G., Coni P., Dessì A., Fraschini M., Fanos V. (2025). They will be famous: Multipotent stem cells in breast milk. World J. Clin. Pediatr..

[16] Park S.B., Yu K.R., Jung J.W., Lee S.R., Roh K.H., Seo M.S., Park J.R., Kang S.K., Lee Y.S., Kang K.S. (2009). BFGF enhances the IGFs-mediated pluripotent and differentiation potentials in multipotent stem cells. Growth Factors.

[17] Kitiyanant N., Kitiyanant Y., Svendsen C.N., Thangnipon W. (2012). BDNF-, IGF-1- and GDNF-Secreting Human Neural Progenitor Cells Rescue Amyloid β-Induced Toxicity in Cultured Rat Septal Neurons. Neurochem. Res..

[18] Youssef A., Aboalola D., Han V.K.M. (2017). The Roles of Insulin-Like Growth Factors in Mesenchymal Stem Cell Niche. Stem Cells Int..

[19] Guo X., Wang C.C., Chung J.P.W., Li T.C., Chen X. (2023). Expression of vascular endothelial growth factor A (VEGFA), placental growth factor (PlGF) and insulin-like growth factor 1 (IGF-1) in serum from women undergoing frozen embryo transfer. Hum. Fertil..

[20] Tamama K., Kawasaki H., Wells A. (2010). Epidermal Growth Factor (EGF) Treatment on Multipotential Stromal Cells (MSCs). Possible Enhancement of Therapeutic Potential of MSC. J. BioMed Biotechnol..

[21] Fang L., Gao Y., Wang Z., Li Y., Yan Y., Wu Z., Cheng J.C., Sun Y.P. (2021). EGF stimulates human trophoblast cell invasion by downregulating ID3-mediated KISS1 expression. Cell Commun. Signal..

[22] Sigismund S., Avanzato D., Lanzetti L. (2018). Emerging functions of the EGFR in cancer. Mol. Oncol..

[23] Tito C., Masciarelli S., Colotti G., Fazi F. (2025). EGF receptor in organ development, tissue homeostasis and regeneration. J. BioMed Sci..

[24] Fei Y., Xiao L., Doetschman T., Coffin D.J., Hurley M.M. (2011). Fibroblast Growth Factor 2 Stimulation of Osteoblast Differentiation and Bone Formation Is Mediated by Modulation of the Wnt Signaling Pathway. J. Biol. Chem..

[25] Fistrek Prlic M., Vukovic Brinar I., Kos J., Dika Z., Ivandic E., Fucek M., Jelaković B. (2024). Serum Hepatocyte Growth Factor Concentration Correlates with Albuminuria in Individuals with Optimal Blood Pressure and Untreated Arterial Hypertension. Biomedicines.

[26] Prlic M.F., Kos J., Fucek M., Knezevic T., Ivkovic V., Jelakovic A., Josipovic J., Simicevic L., Jelakovic B. (2022). Serum Hepatocyte Growth Factor Concentration is Associated with Blood Pressure in Subjects with Prehypertension. J. Hypertens..

[27] Chen D., Zheng J. (2014). Regulation of Placental Angiogenesis. Microcirculation.

[28] Carmeliet P., Moons L., Luttun A., Vincenti V., Compernolle V., De Mol M., Wu Y., Bono F., Devy L., Beck H. (2001). Synergism between vascular endothelial growth factor and placental growth factor contributes to angiogenesis and plasma extravasation in pathological conditions. Nat. Med..

[29] Baraz L.S., Ataca E., Oflas N.D., Kosali S.C., Usta B., Oral A., Cihangiroglu M., Ozgen M., Kehribar D.Y. (2026). Altered NGF and GDNF levels reveal neuroimmune dysregulation in COVID-19 patients. Sci. Rep..

[30] Palasz E., Wilkaniec A., Stanaszek L., Andrzejewska A., Adamczyk A. (2023). Glia-Neurotrophic Factor Relationships: Possible Role in Pathobiology of Neuroinflammation-Related Brain Disorders. Int. J. Mol. Sci..

[31] Huang J., Wang W., Peng Y., Weng W., Wei H., Feng Q., Wang A., Hu M., Li Z., Sun S. (2025). Neurophilic Biomimetic Lipoprotein-Mediated Targeted Nerve Growth Factor Delivery for Traumatic Brain Injury Therapy. Adv. Sci..

[32] Lv J., Xie M., Zhao S., Qiu W., Wang S., Cao M. (2021). Nestin is essential for cellular redox homeostasis and gastric cancer metastasis through the mediation of the Keap1–Nrf2 axis. Cancer Cell Int..

[33] Wang Y., Liao G., Wu Y., Wang R., Tang D.D. (2023). The intermediate filament protein nestin serves as a molecular hub for smooth muscle cytoskeletal signaling. Respir. Res..

[34] Daido W., Nakashima T., Masuda T., Sakamoto S., Yamaguchi K., Horimasu Y., Miyamoto S., Iwamoto H., Fujitaka K., Hamada H. (2023). Nestin and Notch3 collaboratively regulate angiogenesis, collagen production, and endothelial–mesenchymal transition in lung endothelial cells. Cell Commun. Signal..

[35] Kondracka A., Gil-Kulik P., Wojtowicz-Marzec M., Petniak A., Kondracki B., Kwasniewska A., Kocki J. (2023). Expression of microRNA (miR126*, miR155, miR21, miR29a) in breast milk cell fraction in women with hypertension: A comparative analysis with women without hypertension. Ginekol. Pol..

[36] Stanko P., Kaiserova K., Altanerova V., Altaner C. (2014). Comparison of human mesenchymal stem cells derived from dental pulp, bone marrow, adipose tissue, and umbilical cord tissue by gene expression. BioMed Pap..

[37] Secco M., Moreira Y.B., Zucconi E., Vieira N.M., Jazedje T., Muotri A.R., Okamoto O.K., Verjovski-Almeida S., Zatz M. (2009). Gene Expression Profile of Mesenchymal Stem Cells from Paired Umbilical Cord Units: Cord is Different from Blood. Stem Cell Rev. Rep..

[38] Heo J.S., Choi Y., Kim H.S., Kim H.O. (2016). Comparison of molecular profiles of human mesenchymal stem cells derived from bone marrow, umbilical cord blood, placenta and adipose tissue. Int. J. Mol. Med..

[39] Mortazavi Y., Sheikhsaran F., Soleimani M., Khamisipour G., Teimuri A., Shokri S. (2016). The Evaluation of Nerve Growth Factor Over Expression on Neural Lineage Specific Genes in Human Mesenchymal Stem Cells. Cell J..

[40] Hosseini S.M., Talaei-khozani T., Sani M., Owrangi B. (2014). Differentiation of Human Breast-Milk Stem Cells to Neural Stem Cells and Neurons. Neurol. Res. Int..

[41] Guo Z.Y., Sun X., Xu X.L., Peng J., Wang Y. (2015). Human umbilical cord mesenchymal stem cells promote peripheral nerve repair via paracrine mechanisms. Neural Regen. Res..

[42] Chi H., Guan Y., Li F., Chen Z. (2019). The Effect of Human Umbilical Cord Mesenchymal Stromal Cells in Protection of Dopaminergic Neurons from Apoptosis by Reducing Oxidative Stress in the Early Stage of a 6-OHDA-Induced Parkinson’s Disease Model. Cell Transplant..

[43] Ballard O., Morrow A.L. (2013). Human Milk Composition. Pediatr. Clin. N. Am..

[44] Schreier B., Rabe S., Schneider B., Bretschneider M., Rupp S., Ruhs S., Neumann J., Rueckschloss U., Sibilia M., Gotthardt M. (2013). Loss of Epidermal Growth Factor Receptor in Vascular Smooth Muscle Cells and Cardiomyocytes Causes Arterial Hypotension and Cardiac Hypertrophy. Hypertension.

[45] Takayanagi T., Kawai T., Forrester S.J., Obama T., Tsuji T., Fukuda Y., Elliott K.J., Tilley D.G., Davisson R.L., Park J.Y. (2015). Role of Epidermal Growth Factor Receptor and Endoplasmic Reticulum Stress in Vascular Remodeling Induced by Angiotensin II. Hypertension.

[46] Chan S.L., Umesalma S., Baumbach G.L. (2015). Epidermal Growth Factor Receptor Is Critical For Angiotensin II–Mediated Hypertrophy in Cerebral Arterioles. Hypertension.

[47] Lundstam U., Hägg U., Bergmann Sverrisdottir Y., Svensson L.E.T., Gan L.M. (2007). Epidermal growth factor levels are related to diastolic blood pressure and carotid artery stiffness. Scand. Cardiovasc. J..

[48] Al-Jarallah A., Kalakh S., Akhtar S., Yousif M.H.M. (2025). HDL attenuates Ang II–AT1R–EGFR signaling and reverses vascular remodeling in spontaneously hypertensive rats. Front. Pharmacol..

[49] Armant D.R., Fritz R., Kilburn B.A., Kim Y.M., Nien J.K., Maihle N.J., Romero R., Leach R.E. (2015). Reduced expression of the epidermal growth factor signaling system in preeclampsia. Placenta.

[50] Xu Z., Luo W., Chen L., Zhuang Z., Yang D., Qian J., Khan Z.A., Guan X., Wang Y., Li X. (2022). Ang II (Angiotensin II)–Induced FGFR1 (Fibroblast Growth Factor Receptor 1) Activation in Tubular Epithelial Cells Promotes Hypertensive Kidney Fibrosis and Injury. Hypertension.

[51] Barik P., Kuo W.W., Kuo C.H., Hsieh D.J.Y., Day C.H., Daddam J., Chen M.Y.C., Padma V.V., Shibu M.A., Huang C.Y. (2023). Rewiring of IGF1 secretion and enhanced IGF1R signaling induced by co-chaperone carboxyl-terminus of Hsp70 interacting protein in adipose-derived stem cells provide augmented cardioprotection in aging-hypertensive rats. Aging.

[52] Kim H.Y., Moon S., Song K.H., Kim H.J. (2025). Diagnostic applicability of the circulating growth factors in preeclampsia. Sci. Rep..

[53] Medzikovic L., Ruffenach G., Dehghanitafti A., Wong B., Ryder A., Hatamnejad M.R., Sun W., Esdin L., Eghbali J., Brownstein A. (2026). Hepatocyte growth factor may contribute to male protection against pulmonary arterial hypertension. Biol. Sex. Differ..

[54] Qi M., Xin S. (2019). FGF signaling contributes to atherosclerosis by enhancing the inflammatory response in vascular smooth muscle cells. Mol. Med. Rep..

[55] Przywara D., Babiuch W., Petniak A., Wasilewska M., Krzyżanowski J., Czuba M., Krzyżanowski A., Kondracka A., Kocki J., Gil-Kulik P. (2026). Umbilical Cord Blood Gasometry and pH as Key Regulators of Growth Factor Expression Profile in Umbilical Cord-Derived Mesenchymal Stromal Cells (UC-MSCs). Cells.

[56] Chang Y.J., Hwu C.M., Yeh C.C., Wang P., Wang S.W. (2014). Effects of Subacute Hypothyroidism on Metabolism and Growth-Related Molecules. Molecules.

[57] Iglesias P., Bayón C., Méndez J., Gancedo P.G., Grande C., Díez J.J. (2001). Serum Insulin-Like Growth Factor Type 1, Insulin-Like Growth Factor-Binding Protein-1, and Insulin-Like Growth Factor-Binding Protein-3 Concentrations in Patients with Thyroid Dysfunction. Thyroid.

[58] Eke Koyuncu C., Turkmen Yildirmak S., Temizel M., Ozpacaci T., Gunel P., Cakmak M., Ozbanazi Y.G. (2013). Serum Resistin and Insulin-Like Growth Factor-1 Levels in Patients with Hypothyroidism and Hyperthyroidism. J. Thyroid Res..

[59] Agrogiannis G.D., Sifakis S., Patsouris E.S., Konstantinidou A.E. (2014). Insulin-like growth factors in embryonic and fetal growth and skeletal development (Review). Mol. Med. Rep..

[60] Okada M., Kamiya Y., Ito J., Yoshimata T., Kawaguchi M., Shibata H., Fujinami T. (1998). Platelet Epidermal Growth Factor in Thyroid Disorders. Endocr. J..

[61] Davis P., Davis F., Mousa S. (2009). Thyroid Hormone-Induced Angiogenesis. Curr. Cardiol. Rev..

[62] Quadrozzi A., Mayrovitz H.N. (2025). Effects of Thyroid Dysfunction on Angiogenesis During Wound Healing and Skin Repair: A Systematic Review. Cureus.

[63] Paśko P., Kryczyk-Kozioł J., Zagrodzki P., Prochownik E., Ziomek M., Lauterbach R., Huras H., Staśkiewicz M., Dobrowolska-Iwanek J. (2025). Pilot Study of Growth Factors in Colostrum: How Delivery Mode and Maternal Health Impact IGF-1, EGF, NGF, and TGF-β Levels in Polish Women. Nutrients.

[64] Hellström A., Ley D., Hansen-Pupp I., Hallberg B., Löfqvist C., Van Marter L., Van Weissenbruch M., Ramenghi L.A., Beardsall K., Dunger D. (2016). Insulin-like growth factor 1 has multisystem effects on foetal and preterm infant development. Acta Paediatr..

[65] Wang Q., Zhang F., Hong Y. (2018). Blocking of autocrine IGF-1 reduces viability of human umbilical cord mesenchymal stem cells via inhibition of the Akt/Gsk-3β signaling pathway. Mol. Med. Rep..

[66] Huang Y., Qiu R., Mai W., Kuang J., Cai X., Dong Y., Hu Y.Z., Song Y.B., Cai A.P., Jiang Z.G. (2012). Effects of insulin-like growth factor-1 on the properties of mesenchymal stem cells in vitro. J. Zhejiang Univ. Sci. B.

[67] Wang L., Yang M., Jin M., Wu Y., Zheng T., Gu S., Hua X. (2018). Transplant of insulin-like growth factor-1 expressing bone marrow stem cells improves functional regeneration of injured rat uterus by NF -κB pathway. J. Cell Mol. Med..

[68] Zhou C., Lv M., Wang P., Guo C., Ni Z., Bao H., Tang Y., Cai H., Lu J., Deng W. (2021). Sequential activation of uterine epithelial IGF1R by stromal IGF1 and embryonic IGF2 directs normal uterine preparation for embryo implantation. J. Mol. Cell Biol..

[69] O’Kusky J., Ye P. (2012). Neurodevelopmental effects of insulin-like growth factor signaling. Front. Neuroendocrinol..

[70] Qin Y., Meng S., Lyu C., Wang S. (2026). IGF1 is Reduced in Pregnancies with Preeclampsia and its Influence on Biological Behavior of Trophoblast Cells. Biochem. Genet..

[71] Pers Y.M., Bony C., Duroux-Richard I., Bernard L., Maumus M., Assou S., Barry F., Jorgensen C., Noël D. (2021). MiR-155 Contributes to the Immunoregulatory Function of Human Mesenchymal Stem Cells. Front. Immunol..

[72] Schjenken J.E., Moldenhauer L.M., Zhang B., Care A.S., Groome H.M., Chan H.Y., Hope C.M., Barry S.C., Robertson S.A. (2020). MicroRNA miR-155 is required for expansion of regulatory T cells to mediate robust pregnancy tolerance in mice. Mucosal Immunol..

[73] Yang B., Yang R., Xu B., Fu J., Qu X., Li L., Dai M., Tan C., Chen H., Wang X. (2021). MiR-155 and miR-146a collectively regulate meningitic Escherichia coli infection-mediated neuroinflammatory responses. J. Neuroinflammation.

[74] Ma X., Huang T., Chen X., Li Q., Liao M., Fu L., Huang J., Yuan K., Wang Z., Zeng Y. (2025). Molecular mechanisms in liver repair and regeneration: From physiology to therapeutics. Signal Transduct. Target Ther..

[75] Migita K., Iwanaga N., Izumi Y., Kawahara C., Kumagai K., Nakamura T., Koga T., Kawakami A. (2017). TNF-α-induced miR-155 regulates IL-6 signaling in rheumatoid synovial fibroblasts. BMC Res. Notes.

[76] Coudriet G.M., He J., Trucco M., Mars W.M., Piganelli J.D. (2010). Hepatocyte Growth Factor Modulates Interleukin-6 Production in Bone Marrow Derived Macrophages: Implications for Inflammatory Mediated Diseases. PLoS ONE.

[77] Huang Y., Chen J., Zhou Y., Tang S., Li J., Yu X., Mo Y., Wu Y., Zhang Y., Feng Y. (2016). Circulating miR155 expression level is positive with blood pressure parameters: Potential markers of target-organ damage. Clin. Exp. Hypertens..

[78] Salamt N., Che Roos N.A., Mohamed Shakrin N.N.S., Ugusman A. (2026). MicroRNA-155 at the crossroads of inflammation and vascular dysfunction in essential hypertension. Mol. Biol. Rep..

[79] Hubalewska-Dydejczyk A., Trofimiuk-Müldner M., Ruchala M., Lewiński A., Bednarczuk T., Zgliczyński W., Syrenicz A., Kos-Kudla B., Jarząb B., Gietka-Czernel M. (2021). Thyroid diseases in pregnancy: Guidelines of the Polish Society of Endocrinology. Endokrynol. Pol..

[80] Hassiotou F., Beltran A., Chetwynd E., Stuebe A.M., Twigger A.J., Metzger P., Trengove N., Lai C.T., Filgueira L., Blancafort P. (2012). Breastmilk Is a Novel Source of Stem Cells with Multilineage Differentiation Potential. Stem Cells.

[81] Gil-Kulik P., Leśniewski M., Bieńko K., Wójcik M., Więckowska M., Przywara D., Petniak A., Kondracka A., Świstowska M., Szymanowski R. (2023). Influence of Perinatal Factors on Gene Expression of IAPs Family and Main Factors of Pluripotency: OCT4 and SOX2 in Human Breast Milk Stem Cells-A Pre-liminary Report. Int. J. Mol. Sci..

[82] Zarychta J., Kowalczyk A., Słowik K., Przywara D., Petniak A., Kondracka A., Wójtowicz-Marzec M., Słyk-Gulewska P., Kwaśniewska A., Kocki J. (2024). Pilot Study on the Effect of Patient Condition and Clinical Parameters on Hypoxia-Induced Factor Expression: HIF1A, EPAS1 and HIF3A in Human Colostrum Cells. Int. J. Mol. Sci..

[83] Gil-Kulik P., Przywara D., Mitrus M., Petniak A., Kondracki B., Szymanowska U., Szymanowski R., Czuba M., Kondracka A., Kocki J. (2026). Higher expression of SOX1, miR-155, and miR-21 in the colostrum of SARS-CoV-2-infected mothers. Sci. Rep..

[84] Gil-Kulik P., Świstowska M., Krzyżanowski A., Petniak A., Kwaśniewska A., Płachno B.J., Galkowski D., Bogucka-Kocka A., Kocki J. (2022). Evaluation of the Impact of Pregnancy-Associated Factors on the Quality of Wharton’s Jelly-Derived Stem Cells Using SOX2 Gene Expression as a Marker. Int. J. Mol. Sci..

[85] Gil-Kulik P., Świstowska M., Kondracka A., Chomik P., Krzyżanowski A., Kwaśniewska A., Rahnama M., Kocki J. (2020). Increased Expression of BIRC2, BIRC3, and BIRC5 from the IAP Family in Mesenchymal Stem Cells of the Umbilical Cord Wharton’s Jelly (WJSC) in Younger Women Giving Birth Naturally. Oxid. Med. Cell Longev..

[86] Gil-Kulik P., Chomik P., Krzyżanowski A., Radzikowska-Büchner E., Maciejewski R., Kwaśniewska A., Rahnama M., Kocki J. (2019). Influence of the Type of Delivery, Use of Oxytocin, and Maternal Age on POU5F1 Gene Expression in Stem Cells Derived from Wharton’s Jelly within the Umbilical Cord. Oxid. Med. Cell Longev..

[87] Gil-Kulik P., Krzyżanowski A., Dudzińska E., Karwat J., Chomik P., Świstowska M., Kondracka A., Kwaśniewska A., Cioch M., Jojczuk M. (2019). Potential Involvement of BIRC5 in Maintaining Pluripotency and Cell Differentiation of Human Stem Cells. Oxid. Med. Cell Longev..

[88] Walecka I., Gil-Kulik P., Krzyżanowski A., Czop M., Galkowski D., Karwat J., Chomik P., Świstowska M., Kwaśniewska A., Bogucka-Kocka A. (2017). Phenotypic Characterization of Adherent Cells Population CD34+ CD90+ CD105+ Derived from Wharton’s Jelly. Med. Sci. Monit..

[89] Livak K.J., Schmittgen T.D. (2001). Analysis of Relative Gene Expression Data Using Real-Time Quantitative PCR and the 2−ΔΔCT Method. Methods.

[90] Kolberg L., Raudvere U., Kuzmin I., Adler P., Vilo J., Peterson H. (2023). g:Profiler—Interoperable web service for functional enrichment analysis and gene identifier mapping (2023 update). Nucleic Acids Res..

