# Supplementary material for: Maternal Endocrine and Vascular Perturbations Reprogram the Transcriptomic and Secretory Profile of Perinatal Stem Cells: A Comparative Study of Umbilical Cord-Derived Stem Cell UC-MSCs and Human Milk-Derived Stem Cell MM-MSCs
Source: Int J Mol Sci. 2026 Sep 17;27(18):8269. doi: 10.3390/ijms27188269 (PMC13607156; doi:10.3390/ijms27188269)
Supplement: Supplementary file 1 [file ijms-27-08269-s001.zip › ijms-4423362-supplementary.pdf]

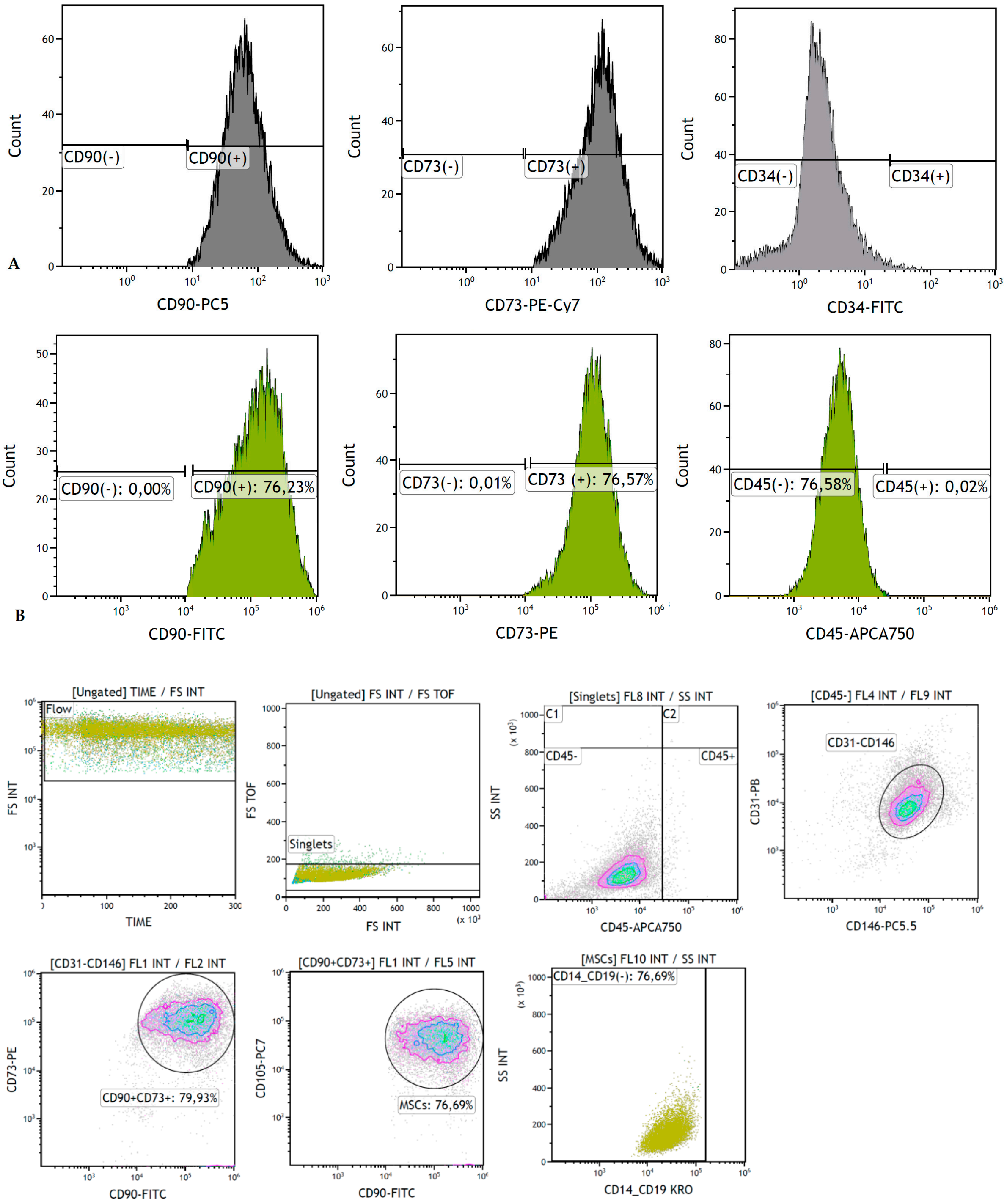

Supplementary Figure S1. Flow cytometry immunophenotypic characterization of isolated perinatal mesenchymal stem cells. Representative histograms confirming the phenotypic identity of the cultured cells in accordance with the International Society for Cell & Gene Therapy (ISCT) criteria. **A)** UC-MSCs: The analyzed cell population demonstrates high, uniform expression of characteristic positive surface markers, including CD90 and CD73. Concurrently, the cells are definitively negative for the hematopoietic lineage marker CD34.

B) MM-MSCs: The isolated lactation stem cell fraction exhibits strong positive expression for the classic mesenchymal stromal markers CD90 and CD73. Crucially, the functional purity of the isolation is confirmed by the lack of expression of the leukocyte common antigen CD45.

A sequential gating strategy was applied to ensure high sample purity and to exclude non-mesenchymal cell contaminants. First, singlet events were identified by gating based on forward scatter intensity (FS INT) versus either time or forward scatter peak/time of flight (FS Peak/FS TOF), thereby excluding doublets and cellular aggregates. Cell type-specific gating strategies were subsequently applied. For Wharton's jelly-derived MSCs (UC-MSCs), cells were initially identified based on the co-expression of the classical mesenchymal markers CD90 and CD73. Within this population, a negative gate was applied to confirm the absence of the hematopoietic marker CD34 ($CD34^-$). For MM-MSCs, analysis was first restricted to singlets. Hematopoietic contamination was then excluded by gating on the $CD45^-$ population. Within this non-hematopoietic fraction, the perivascular mesenchymal profile was isolated by confirming CD146 expression and the absence of the endothelial marker CD31 ($CD31^-$CD146+). Finally, the mesenchymal stromal cell phenotype was confirmed by identifying the CD90+CD73+ and CD105+ populations, followed by verifying the absence of additional lineage markers, including CD14/CD19 ($CD14^-$/$CD19^-$) and CD34.
